# Complement Factor H Is an ICOS Ligand Modulating Tregs in the Glioma Microenvironment

**DOI:** 10.1158/2326-6066.CIR-23-1092

**Published:** 2024-10-08

**Authors:** Karolina I. Smolag, Jakub Olszowka, Rebecca Rosberg, Elinn Johansson, Elisabet Marinko, Karin Leandersson, David J. O’Connell, Valeria Governa, Emre Can Tuysuz, Mattias Belting, Alexander Pietras, Myriam Martin, Anna M. Blom

**Affiliations:** 1 Section of Medical Protein Chemistry, Department of Translational Medicine, Lund University, Malmö, Sweden.; 2 Division of Translational Cancer Research, Department of Laboratory Medicine, Lund University, Lund, Sweden.; 3 Cancer Immunology, Department of Translational Medicine, Lund University, Malmö, Sweden.; 4 School of Biomolecular and Biomedical Science, Conway Institute, University College Dublin, Dublin, Ireland.; 5 Division of Oncology, Department of Clinical Sciences, Lund University, Lund, Sweden.

## Abstract

The survival rate of patients with glioma has not significantly increased in recent years despite aggressive treatment and advances in immunotherapy. The limited response to treatments is partially attributed to the immunosuppressive tumor microenvironment, in which regulatory T cells (Treg) play a pivotal role in immunologic tolerance. In this study, we investigated the impact of complement factor H (FH) on Tregs within the glioma microenvironment and found that FH is an ICOS ligand. The binding of FH to this immune checkpoint molecule promoted the survival and function of Tregs and induced the secretion of TGFβ and IL10 while suppressing T-cell proliferation. We further demonstrated that cancer cells in human and mouse gliomas directly produce FH. Database investigations revealed that upregulation of FH expression was associated with the presence of Tregs and correlated with worse prognosis for patients with glioma. We confirmed the effect of FH on glioma development in a mouse model, in which FH knockdown was associated with a decrease in the number of ICOS^+^ Tregs and demonstrated a tendency of prolonged survival (*P* = 0.064). Because the accumulation of Tregs represents a promising prognostic and therapeutic target, evaluating FH expression should be considered when assessing the effectiveness of and resistance to immunotherapies against glioma.

## Teaser

This study identifies complement factor H as a ligand for the immune checkpoint molecule ICOS, which impacts Treg function in glioma. Targeting FH could improve immunotherapy, offering new strategies for treating this immunosuppressive cancer.

## Introduction

Gliomas are the most common type of malignant primary intracranial tumor among adults. Despite the aggressive standard of care involving surgical resection, radiotherapy, and temozolomide chemotherapy, the prognosis for patients with this malignancy remains poor (1). The unprecedented success of immune checkpoint inhibitors in other types of cancers has given rise to new hope for glioma treatment. However, so far, despite discoveries of several immune targets for immunotherapy, no benefit of inhibiting immune checkpoints in glioma has been proven.

One explanation for poor antitumoral outcomes is the intratumoral and systemic immunosuppression induced by gliomas (2–4). Accumulation of regulatory T cells (Treg) in tumors is a crucial aspect of local immune dysfunction. Although rarely present in normal brain tissue (5), the number of Tregs in animal models increases within 10 days of tumor implementation (6, 7), which correlates with impaired responsiveness of effector T cells (3) and worse survival of patients with glioma (8).

ICOS is an important immune cell costimulatory molecule. The engagement of the only known ligand of ICOS (ICOSL), facilitates a series of immune-related processes in the microenvironment, but the main function is an effect on T cells themselves (9). Because ICOS is expressed on both Tregs and cytotoxic T cells, it can play a dual role in cancer progression. On the one hand, ICOS significantly promotes Treg functions, including survival, differentiation, and proliferation, as well as upregulation of cytokine secretion, including IL10, consequently facilitating immune escape (10, 11). On the other hand, this receptor exerts an antitumor effect through the enhancement of effector T cells (12, 13). Thus, it is not surprising that studies seeking to determine the role of ICOS in different types of tumors report contradictory results, and both ICOS agonists and antagonists are investigated in currently ongoing clinical trials (14–17).

The complement system plays a crucial role in innate immune defense by the elimination of pathogens and modified host cells, and it acts as a vital bridge to the adaptive immune system. Complement regulators safeguard cells and tissues against accidental damage caused by the complement system. Complement factor H (FH), a key inhibitor, is vital for the control of the alternative complement pathway. It accelerates the decay of the alternative pathway C3 convertase and serves as a cofactor for the factor I-mediated cleavage and inactivation of C3b. Absence of FH results in uncontrolled activation of the alternative complement pathway. The FH molecule consists of 20 complement control protein (CCP) modules, each linked by short connectors and arranged sequentially (18, 19). The first four CCPs, along with the 19th and 20th, bind to specific regions of C3b. CCPs 6 to 8 and CCPs 19 to 20 interact with host surfaces (20). Additionally, FH exhibits many noncanonical roles, including differentiation and polarization of infiltrating monocytes into tumor-supporting macrophages (21). Produced mainly in the liver, FH can also be expressed by various cell types, including lung (22) and ovarian cancer cell lines (23). In our recent report, we also described FH expression in human breast cancer (24).

Here, we demonstrate that plasma-purified and glioma cell-derived FH increased the viability of Tregs by binding to ICOS. Furthermore, we propose that this phenomenon is important in glioma as evidenced by our findings that higher FH expression correlated with a worse prognosis and an increased presence of Tregs in both murine and human tumors.

## Materials and Methods

### Study approval

Clinical specimens were collected from patients with glioma (World Health Organization grades II to IV) referred to the Neurosurgery Department at Lund University Hospital. The study was carried out according to the ICH/GCP guidelines, in agreement with the Helsinki Declaration, and approved by the Regional Ethical Review Board in Lund (permit Dnr. 2018/37). Peripheral blood for isolation of peripheral blood mononuclear cells (PBMCs) was obtained from healthy volunteers according to the Declaration of Helsinki and approved by the Regional Ethical Review Board in Lund (permit Dnr. 2017/582). All participants provided informed written consent to participate in the study. The animal studies were approved by the Malmö-Lund Ethical Committee (Dnr. M-16123/19), and the use of mice was conducted following the European Union directive on animal rights.

### Isolation and activation of primary cells

PBMCs were purified by density gradient centrifugation over LymphoPrep (#1114545; Axis-Shield). CD4^+^ T cells were isolated from PBMCs using CD4 MicroBeads (#130-045-101; Miltenyi Biotech). CD8^+^ T cells were isolated using CD8 MicroBeads (#130-045-201; Miltenyi Biotech). Naïve T cells were isolated using Naïve CD4^+^ T-cell Isolation Kit II (#130-094-131; Miltenyi Biotech). Tregs were isolated using CD4^+^CD25^+^CD127^dim/−^ Regulatory T-cell Isolation Kit (#130-094-775; Miltenyi Biotech). The CD4^+^(−Tregs) cells were isolated from CD4^+^ T cells by depleting Tregs with CD4^+^CD25^+^CD127^dim/−^ Regulatory T-cell Isolation Kit. T cells were cultured in TexMACS medium (#130-097-196; Miltenyi Biotech) with 20 IU (CD4^+^, CD8^+^, and naïve T cells) or 500 IU IL2 (Tregs; #130-097-743; Miltenyi Biotech). CD4^+^ T cells were activated using TransAct (#130-111-160; Miltenyi Biotech) or TransAct with staphylococcal enterotoxin A (SEA; #S9399; Sigma-Aldrich) according to the manufacturer’s instructions. All incubations were performed at a temperature of 37°C and a CO_2_ concentration of 5%. Unless stated otherwise, cells were stimulated with 150 μg/mL FH or alpha-1 antitrypsin (α1-AT) as a negative control.

### Cell lines

DF-1 (ATCC, Cat. # CRL-12203, RRID:CVCL_0570) cells were acquired from ATCC in 2015, and H4 (ATCC, Cat. # CRL-1600, RRID:CVCL_0285) cells were acquired from ATCC in 2020. NIH3T3-tva cells were kindly provided by Eric Holland (Fred Hutchinson Cancer Center). The H4 glioma cells were stably transfected with FH in pCMV3 or empty pCMV3 vector (Sino Biological). Plartelet-derived growth factor B–induced glioma primary culture (PIGPC) cells (25) were isolated from Ntv-a mice using an adult brain dissociation kit (#130-107-677; Miltenyi) and cultured in PIGPC medium containing neurobasal medium (#21103049; Life Technologies) mixed with an equal volume of DMEM/F12 with Glutamax (#31331028; Life Technologies) and supplemented with N2 supplement (#15410294; Gibco), B27 supplement (#11500446; Fisher Scientific), EGF (#AF-100-15; Peprotech), and basic fibroblast growth factor (#100-18B; Peprotech). Cell lines tested negative for mycoplasma in a routine test performed every 2 months (H4) or quarterly (NIH3T3-tva, DF-1) and were not authenticated after they were obtained. Mycoplasma tests were RT-PCR based and were done by GATC Biotech (Eurofins). All incubations were performed at a temperature of 37°C and a CO_2_ concentration of 5%.

### Proteins

FH and α1-AT were purified from human plasma as described before (26, 27). In brief, proteins with higher molecular weights were removed from the plasma using 15% ammonium sulfate. The fraction of the supernatant containing FH was precipitated using 40% ammonium sulfate. FH was then purified from this fraction using an affinity column with immobilized monoclonal MRC-OX24 anti-FH (purity based on Western blot analysis at least 95%). α1-AT was purified using the Äkta Explorer 100 automated chromatography system (GE Healthcare). Thiol proteins initially attach to matrix-associated activated thiol compounds through an SH–SS exchange reaction. Subsequently, the mixed disulfides underwent a two-step reduction process and were further separated by being passed through Blue-Sepharose and AH-Sepharose columns. α1-AT was recovered with 95% purity (based on Western blot analysis). Contamination with LPS was tested using a Limulus amebocyte lysate Endochrome-K assay (#R1708K; Charles River). In the working concentration of FH, levels of endotoxin were determined to be <0.01 to 0.03 EU/mL and <0.04 EU/mL for α1-AT. FH fragment CCP19-20 was purified as described (20) and it was kindly provided by Prof. Paul Barlow. In short, the DNA sequences encoding the CCP19-20 segment of FH, obtained from NCBI-GeneBank, were cloned into the expression vector pPICZα (Pichia pastoris, Invitrogen). Expressed proteins were targeted to the secretory pathway by positioning the coding sequence behind the Saccharomyces cerevisiae α-mating factor secretion sequence. Protein was expressed following transformation by electroporation into P. pastoris strain KM71H (Invitrogen). The initial purification stage involved cation- or anion-exchange chromatography, followed by gel-filtration chromatography. Mouse FH was acquired from Bio-techne (#4999-FH-050), and the extracellular domain of ICOSL was obtained from Abcam (#ab214138). Proteins were labeled with Alexa Fluor 647 (#A20173; Thermo Fisher Scientific) or Alexa Fluor 488 (#A10235; Thermo Fisher Scientific) according to the manufacturer’s instructions. Proteins were biotinylated using an EZ-Link Sulfo-NHS-LC-Biotinylation kit (#1854210; Thermo Fisher Scientific) according to the manufacturer’s instructions. Denatured FH (dFH) was prepared by incubation of FH for 30 minutes at 96°C.

### Generation of FH-depleted supernatants

FH-transfected H4 cells were cultured in DMEM (#SH30243.01; HyClone) with 10% FBS (#30-2020; ATCC), washed four times with DMEM without FBS, and cultured in DMEM without FBS to remove all serum from the supernatant. After 24 hours, cells were washed once and seeded in DMEM without FBS. After 4 days, cells were collected and centrifuged at 16,000 × *g* to remove cells and debris. FH was partially depleted from supernatants using protein G Dynabeads (#100.03D; Thermo Fisher Scientific) coupled with MRC-OX24 anti-FH antibody (ECACC Hybridoma Collection). After 24 hours of incubation, the beads were removed by centrifugation.

### Binding of FH to T cells

Human CD4^+^ and CD8^+^ T cells and mouse Tregs were incubated in medium only or medium supplemented with 50 or 100 μg/mL biotinylated human FH, or 25 or 100 μg/mL Alexa Fluor 488-labeled mouse FH for 2 hours at 37°C. Bound biotinylated human FH was detected with Alexa Flour 647-labeled streptavidin (#17-4317-82; Life Technologies) and the binding of human and mouse FH was measured in a CytoFLEX flow cytometer (Beckman Coulter). Analysis of the results was performed with FlowJo software (Tree Star, RRID:SCR_008520), version 9. Gating strategies for flow cytometry are shown in the supplementary figures (Supplementary Fig. S1I)

### Cell fractionation

CD4^+^ and CD8^+^ T cells were incubated with biotinylated human FH for 2 hours at 37°C. Cytosolic and membrane/organelle fractions were isolated using Mem-Per plus eukaryotic protein extraction kit (#89842; Thermo Fisher Scientific), according to the manufacturer’s instructions. Proteins in each fraction were reduced in SDS-PAGE loading buffer, separated on 10% SDS-PAGE, transferred to a membrane and detected by Western blot using goat anti-human FH antiserum (Quidel, Cat. # A312, RRID:AB_452513) and antibodies for GAPDH (Abcam, Cat. # ab8245, RRID:AB_2107448) and Na^+^K^+^ATPase (Abcam, Cat. # ab8245, RRID:AB_2107448), followed by polyclonal swine anti-rabbit (Agilent, Cat. # P0399, RRID:AB_2617141), goat anti-mouse (Agilent, Cat. # P0447, RRID:AB_2617137), and rabbit anti-goat (Agilent, Cat. # P0449, RRID:AB_2617143) IgG/horseradish peroxidase (HRP). Proteins were visualized using ChemiDoc MP system (Bio-Rad) with 3,3′,5,5′-tetramethylbenzidine (TMB) substrate (#4380A; ECO-TEK).

### Identification of binding partners for FH

For protein microarray, HuProt human protein microarray slides (CDI Labs, v4) were blocked for 90 minutes in buffer [50-mmol/L 4-(2-hydroxyethyl)-1-piperazineethanesulfonic acid, pH 7.5, 25% glycerol, 200-mmol/L NaCl, 1% milk, 0.08% Triton X-100, 1-mmol/L dithiothreitol, and 20-mmol/L reduced glutathione]. Alexa Fluor 647-labeled FH was diluted to 500 nmol/L in washing buffer (PBS, pH 7.4, 1% milk, and 0.1% Tween 20) and added onto the slides for 1 hour at 4°C. The slides were washed five times in washing buffer and the signal was read in a GenePix 4000B series scanner GenePix Pro software was used to determine specific binding partners.

### Binding of FH to ICOS

For co-immunoprecipitation, CD4^+^ T cells were incubated with human FH or biotinylated FH. Cells were lysed with lysis buffer [150 mmol/L NaCl, 50 mmol/L Tris-HCl, pH 7.5, 1% NP40, 0.1% SDS, 0.5% deoxycholate, and HALT Protease and Phosphatase inhibitor cocktail (#78420; Thermo Fisher Scientific)], and the protein complexes in the lysates were pulled down with streptavidin magnetic beads (#88817; Pierce). The presence of ICOS in the pulled-down protein complexes was detected with anti-ICOS (Abcam, Cat. # ab183870, RRID:AB_3096167) using Western blotting as described above for FH.

For a proximity ligation assay (PLA), activated and naïve CD4^+^ T cells were incubated with FH and α1-AT for 2 hours at 37°C, washed five times with PBS, and attached to UltraFrost microscopy slides (Thermo Scientific) using a Cellspin centrifuge (Tharmack). Goat anti-human FH antiserum and mouse anti-human ICOS (BD Biosciences, Cat. # 557801, RRID:AB_396877) were used to detect the targets. PLA was performed using DuoLink system (Sigma) according to the manufacturer’s instructions. Spots were imaged by confocal microscope using 63× objective with oil and Zen software (Zeiss).

For FH–ICOS binding assay, CD4^+^ T cells were incubated with or without biotinylated human FH or biotinylated α1-AT for 2 hours at 37°C and lysed with lysis buffer (150 mmol/L NaCl, 50 mmol/L Tris-HCl, pH 7.5, 1% NP40, 0.1% SDS, 0.5% deoxycholate, and HALT Protease and Phosphatase inhibitor cocktail). Microtiter plates were coated with anti-ICOS (Abcam, Cat #. ab183870, RRID:AB_3096167) at room temperature overnight. After 1-hour blocking in quench buffer [3% fish gelatin (#HP-03; Norland Products) in immunowash (50 mmol/L Tris-HCl, 150 mmol/L NaCl, 0.1% Tween 20, and pH 8.0), samples were applied and incubated for 2 hours at 37°C. Biotinylated FH was detected using streptavidin–HRP (#S911; Invitrogen). Bound protein was visualized with TMB substrate (#4380A; ECO-TEK) and an image captured using ChemiDoc MP system (Bio-Rad).

### ICOS blocking

Tregs were incubated for 1 hour at 4°C in medium only, with 1, 2, 5, or 10 μg/mL of ICOS blocking antibody (BioLegend, Cat. # 313502, RRID:AB_416326), or the same concentrations of IgG isotype control (BioLegend, Cat. # 400901, RRID:AB_3096169). Next, cells were treated with 150 μg/mL of biotinylated human FH for 2 hours at 37°C. Bound FH was detected with Alexa Fluor 488-conjugated streptavidin (#12-4317-87; eBioscience) using CytoFLEX flow cytometer (Beckman Coulter) and analyzed with FlowJo software, version 9.

### Dissociation constant (KD) of ICOS ligands

Tregs (1 × 10^5^) were incubated for 2 hours at 4°C in PBS with various concentrations of fluorescently labeled ICOSL (0, 3.3, 7.6, 15.2, 30.3, 60.6, and 121.2 nmol/L) or FH (0, 6.7, 13.3, 20, 26.7, 33.3, 66.7, and 133 mmol/L). Competition assay was performed by preincubation of 1 × 10^5^ Tregs for 2 hours at 4°C with 0, 133 pmol/L of FH or 0, 12,1 pmol/L of ICOSL. The binding of fluorescently labeled 3.3 nmol/L of ICOSL and 13.3 nmol/L of FH to the cells was measured using flow cytometry. Using CytoFLEX flow cytometer (Beckman Coulter) and analyzed with FlowJo software, version 9. Kd was calculated using GraphPad Prism (RRID:SCR_002798), version 8.0.

### PI3K Western blot analysis and inhibition

For PI3K activation, Tregs were pretreated with 5 μg/mL ICOS blocking antibody (BioLegend, Cat. # 313502, RRID:AB_416326) or IgG isotype control (BioLegend, Cat. # 400901, RRID:AB_3096169) for 30 minutes at 37°C. Next, cells were activated with TransAct according to the manufacturer’s instructions and incubated with 150 μg/mL of FH for 15 minutes at 37°C and then washed twice with PBS and finally lysed in NP-40 lysis buffer (50 mmol/L Tris pH 7.4, 50 mmol/L sodium chloride, and 1.0% NP-40) with HALT Protease and Phosphatase Inhibitor Cocktail. Supernatants were cleared from cellular debris by centrifugation. Total protein concentration was measured using the BCA protein assay reagent kit (#23227; Thermo Fisher Scientific). Proteins were separated by gel electrophoresis under reducing conditions and blotted onto a membrane. Anti-Akt (Cell Signaling Technology, Cat. # 4691, RRID:AB_915783), anti-phospho-Akt (Cell Signaling Technology, Cat. # 13038, RRID:AB_2629447), anti-phospho-GSK (Cell Signaling Technology, Cat. # 9331, RRID:AB_329830), and anti-GAPDH (Abcam, Cat. # ab8245, RRID:AB_2107448) primary antibodies, followed by polyclonal swine anti-rabbit (Agilent, Cat. # P0399, RRID:AB_2617141) and goat anti-mouse (Agilent, Cat. # P0447, RRID:AB_2617137) IgG/HRP, were used for immunodetection. Proteins were visualized using ChemiDoc MP system (Bio-Rad) with TMB substrate (#4380A; ECO-TEK). The signal was quantified using Image Lab (RRID:SCR_014210).

### Viability

CD4^+^, CD8^+^, and CD4^+^ naïve T cells, Tregs, CD4^+^(−Tregs), Tregs pretreated with anti-ICOS (BioLegend, Cat. # 313502, RRID:AB_416326), Tregs pretreated with IgG isotype control (BioLegend, Cat. # 400901, RRID:AB_3096169), and Tregs treated with 0.05 or 0.1 μmol/L CAL-101 (#S2226; Selleckchem) were incubated in medium only or medium supplemented with FH, α1-AT, supernatant and partially FH-depleted supernatant from glioma cell lines, prepared as described above, for 7 days. The viability of the various T cell populations was studied by flow cytometry following staining with Annexin V-APC (#31490016X2; ImmunoTools) and 7-AAD Via-Probe (#555815; BD Bioscience). The signal was measured in a CytoFLEX flow cytometer (Beckman Coulter). Analysis of the results was done with FlowJo software version 9. Based on the staining, cells were gated into three populations: live (Annexin V^−^, Via-Probe^−^), early apoptotic (Annexin V^+^, Via-Probe^−^), and Annexin V^+^, Via-Probe^+^.

To detect ICOS dependence, Tregs were incubated in medium only, or medium with FH or α1-AT for 7 days. ICOS on the cell surface was detected with anti-ICOS labeled with Alexa Fluor 647 (BD Biosciences, Cat. # 565882, RRID:AB_2744479). Cells were gated based on the ICOS expression and viability of each group was assessed with SYTOX Green (#S7020; Invitrogen). The signal was measured in CytoFLEX flow cytometer and analyzed with FlowJo software, version 9.

### Immunosuppressive assay

Tregs were pretreated with medium only or supplemented with 150 μg/mL FH for 2 days and cocultured with responder CD4^+^ T cells (Tresp), isolated from the same donor, at different ratios, and stimulated with CD2/CD3/CD28 MACSiBeads (#130-091-441; Miltenyi Biotech) for 5 days. Next, ^3^H-thymidine (#NET027005MC; Perkin Elmer) was added for 16 hours. Tresp alone, Tresp without stimulation, and Tregs without stimulation were used as a control. Thymidine incorporation was measured on Wizard^2^ with gamma counter (Perkin Elmer).

### Cytokine detection

Tregs were stimulated with TransAct according to the manufacturer’s instructions, in combination with 150 μg/mL of FH, α1-AT, and dFH. Supernatants were collected after 24 hours and analyzed with IL10 Max ELISA (#430604; BioLegend) and TGFβ DuoSet ELISA (#DY240-05; R&D). Absorbance measurements were collected using the Infinite 200 PRO microplate reader (Tecan) and analyzed using Microsoft Excel (Microsoft) version 15.29.1.

Alternatively, cells were collected after 16 hours, labeled with anti-ICOS-Alexa Fluor 488 antibody (BD) and IL10 release was detected using IL10 catch (#120-000-868; Miltenyi Biotech) according to the manufacturer’s instructions. Dead cells were excluded based on the forward/side scatter characteristics.

### Proliferation assay

H4 and PIGPC cells were seeded onto a black-sided 96-well plate at a density of 2,000 cells/well in DMEM with 2% FBS (H4 cells) or PIGPC medium described above (PIGPC cells) in triplicates. Plates used for the proliferation of PIGPC cells were ornithine- and laminin-coated. CyQUANT Direct Cell Proliferation Assay (#C35013; Fisher Scientific) was used to detect the proliferation rate of the cells according to the manufacturer’s instructions. Viability was measured at 24, 48, 72, and 96 hours. Data obtained at 24 hours were used for the normalization.

### Generation of murine glioma with FH knockdown

Murine gliomas were generated as described before (28, 29). In short, low-grade gliomas were generated using the RCAS/tv-a vectors to induce expression of platelete-derived growth factor B (PDGFB), whereas *PDGFB* and *-shp53* were used for induction of glioblastoma (GBM). RCAS-shRNA vectors were prepared as described before (30). FH was knocked down in the high-grade glioma model using shRNA Cfh (5′-CCA​TCC​AGG​ATA​TGT​CGG​AAA-3′) or, as a control, shRNA Gl2 (5′-CGT​ACG​CGG​AAT​ACT​TCG​A-3′) (TAG Copenhagen).

DF-1 cells were transfected with vectors using x-tremeGene 9 reagent (#6365779001; Roche); 5 × 10^4^ cells were intracranially injected into the left hemisphere of neonatal Nestin tv-a mice (The Jackson Laboratory; RRID:IMSR_JAX:003529).

NIH3T3-tva cells were transfected using gateway LR Clonase (#11791-019, Invitrogen) with the RCAS-shFH/RCAS-shGl2, and FH secretion was detected by Western blot using anti-mouse FH antibody (Hycult Biotech, Cat. # HM1119, RRID:AB_10679038) followed by goat anti-mouse (Agilent, Cat. # P0447, RRID:AB_2617137) IgG/HRP. Proteins were visualized using ChemiDoc MP system (Bio-Rad) with TMB substrate (#4380A; ECO-TEK).

### Immunofluorescent staining of murine tumors

Upon glioma symptoms, animals were euthanized, and brains were embedded in optimal cutting temperature compound (#45830; Thermo Fisher Scientific) and snap-frozen in ice-cold isopentane. Cryosections were air-dried for 30 minutes following ice-cold acetone fixation. Blocking and permeabilization were performed with 0.5% Triton X-100 in PBS with 1% BSA for 20 minutes, followed by incubation in blocking solution overnight at 4°C with the following primary antibodies: FH (Santa Cruz Biotechnology, Cat. # sc-17951, RRID:AB_2260590), GFAP (Abcam, Cat. # ab4674, RRID:AB_304558), Olig2 (R and D Systems, Cat. # AF2418, RRID:AB_2157554), F4/80 (BioLegend, Cat. # 123117, RRID:AB_893489), CD4 (Abcam, Cat. # ab183685, RRID:AB_2686917), CD8-Alexa 555 (Abcam, Cat. # ab280863, RRID:AB_2927548), FoxP3 (Thermo Fisher Scientific, Cat. # 14-5773-82, RRID:AB_467576), Ki67 (Abcam, Cat. # ab156956, RRID:AB_2732028), ICOS (Cell Signaling Technology, Cat. # 67223, RRID:AB_3096171), and goat IgG (R and D Systems, Cat. # AB-108-C, RRID:AB_354267). After PBS washes, appropriate species-specific, fluorophore-conjugated secondary antibodies were used for staining detection anti-rabbit IgG (Abcam, Cat. # ab150064, RRID:AB_2734146; and Abcam, Cat. # ab150075, RRID:AB_2752244), anti-goat IgG (Abcam, Cat. # ab175745, RRID:AB_2924800; and Abcam, Cat. # ab150134, RRID:AB_2715537), anti-rat IgG (Abcam, Cat. # ab150155, RRID:AB_2813835; and Abcam, Cat. # ab150153, RRID:AB_2737355), and anti-chicken IgY (Abcam, Cat. # ab63507, RRID:AB_1139472). For all stainings, cell nuclei were detected using 4′,6-diamidino-2-phenylindole (DAPI; #D9542; Sigma-Aldrich) and mounted using Diamond ProLong Diamond Antifade Mountant (#P36970; Thermo Fisher Scientific). Tissues were incubated with isotype control (R and D Systems, Cat. # AB-108-C, RRID:AB_354267) or secondary antibodies alone (Abcam, Cat. # ab150134, RRID:AB_2715537) to control for nonspecific signals. The number of ICOS^+^ Tregs is an average number of cells calculated based on measurements from three tumor sections because of the low number of cells and uneven distribution in the tissue. The number of other cell types was calculated based on one slide per tumor. Images were captured using PhenoImager HT (Akoya Biosciences). For each fluorophore, a spectral library was built from corresponding monostaining, to allow for spectral unmixing and autofluorescence removal using inForm software package (RRID:SCR_019155). Cell detection, segmentation, and positive cell detection were performed in QuPath. Cells were characterized from the DAPI channel, followed by classifiers for each marker based on machine learning.

### FH staining in human glioma

Fresh samples of macroscopically viable tumors were directly cryopreserved by snap freezing in isopentane for immunofluorescence evaluation. Human brain tumor cryosections (8 μm) were fixed in 4% paraformaldehyde and permeabilized in 0.5% Triton X-100. Human tumor sections were blocked in 5% donkey serum (#017-000-121; Jackson ImmunoResearch). Anti-FH antibody (Quidel) diluted 1:250 in 5% donkey serum was added overnight at 4°C, followed by incubation with donkey anti-goat Alexa Fluor488-conjugated secondary antibody (Thermo Fisher Scientific, Cat. # A-11055, RRID:AB_2534102) for 1 hour at 4°C. Slices were mounted with a fluorescence mounting medium (#S3023; Dako) and acquired with an LSM 710 confocal microscope equipped with Airyscan array detector unit (Carl Zeiss). Plan-Apochromat 40×/1.40 differential interference contrast M27 oil immersion objective was used. Additionally, the slices were also acquired with a Zeiss\Axio Scan. Z1. Images were processed using ZEN 2.1 Black Edition (RRID:SCR_018163).

### Statistics

Statistical analyses were performed in GraphPad Prism (RRID:SCR_002798), version 8.0, or SPSS Statistics (RRID:SCR_002865) version 23.0. Data are presented as mean ± *SD* and analyzed with one-way ANOVA, two-way ANOVA with Tukey’s multiple comparison test, two-way ANOVA with Bonferroni’s multiple comparison test, Kruskal–Wallis with Dunn’s multiple comparison test, logrank test, Student *t* test with Welch correction, *χ*^2^ test, Spearman’s rho correlation, one-sided Fisher exact test, or Benjamini–Hochberg procedure. *P* and *Q* values of <0.05 are denoted statistically significant. *P* values are displayed as follows: *, *P* < 0.05; **, *P* < 0.01; ***, *P* < 0.001; ****, *P* < 0.0001.

### Analysis of publicly available datasets

Correlation between FH, ICOS, and ICOSL expression and patient survival and other clinical parameters was assessed using cBioPortal (www.cbioportal.org), a comprehensive web resource using The Cancer Genome Atlas (TCGA) database, for visualizing and analyzing multidimensional cancer genomics data (31). Samples derived from 530 patients from the brain lower-grade glioma (TCGA Firehose Legacy https://datacatalog.mskcc.org/dataset/10469) database were analyzed.

Infiltration of Tregs was investigated using TIMER2.0 (https://cistrome.shinyapps.io/timer/; ref. 32). In our study, “Gene module” was used to evaluate the correlation between FH expression and the infiltration of immune cells in lower-grade glioma (*n* = 516) and GBM (*n* = 153).

Expression of FH and Ki67 in tumor cells was determined in 16 patients with GBM. Data was obtained from the Gene Expression Omnibus (GEO) database (https://www.ncbi.nlm.nih.gov/geo/query/acc.cgi?acc=GSE90604; ref. 33). Correlation analyses were performed in GraphPad Prism (RRID:SCR_002798), version 8.0.

### Data availability

The data generated in this study are available within the article and its supplementary data files or from the corresponding author upon reasonable request.

## Results

### FH binds to ICOS on the surface of T cells

**Figure 1.**
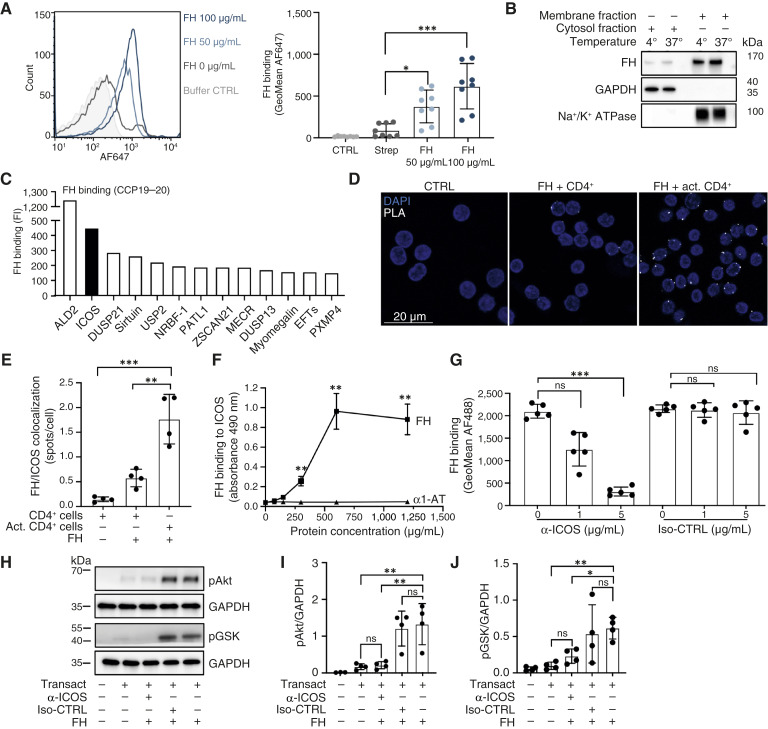
FH binds to T cells via ICOS. **A,** Biotinylated FH binds to CD4^+^ T cells upon incubation for 2 hours with TexMACS medium alone and supplemented with 50 and 100 μg/mL FH. **B,** Fractionation detecting FH binding but not internalization into CD4^+^ T cells, incubated with 150 μg/mL FH for 2 hours at 4°C or 37°C. **C,** HuProt microarray showing top-specific proteins binding FH CCP19-20. **D** and **E,** PLA showing FH binding to ICOS. CD4^+^ T cells and activated CD4^+^ T cells were incubated with FH for 2 hours at 37°C, and the interaction between ICOS and FH was detected with specific antibodies and visualized as white dots. **F,** ELISA detection of dose-dependent binding of FH to ICOS or α1-AT. CD4^+^ T cells were incubated for 2 hours at 37°C with biotinylated FH or α1-AT and lysed. The microtiter plate was coated with an anti-ICOS antibody, and bound complexes from the lysates were detected with streptavidin. **G,** Preincubation with anti-ICOS antibody reduced FH binding to CD4^+^ T cells in a dose-dependent manner. Isotype control was used at the same concentration, without affecting the binding. Representative blot (**H**) and quantification (**I** and **J**) of increased phosphorylation of Akt and GSK in Tregs upon incubation with 150 μg/mL FH. ICOS blocking antibody but not isotype control inhibited this effect. Data are means ± SD of *n* = 8 (**A**), *n* = 5 (**E–G** Iso-CTRL), *n* = 7 (**G** α-ICOS), *n* = 4 (**I** and **J**) independent experiments; representative blot of (**B** and **H**) *n* = 4 independent experiments; representative pictures of (**D**) *n* = 5 independent experiments. Statistical test: Kruskal–Wallis with Dunn’s multiple comparison test (**A** and **E**), two-way ANOVA (**F** and **G**), and one-way ANOVA (**I** and **J**) with Tukey’s multiple comparison test. *, *P* < 0.05; **, *P* < 0.01; ***, *P* < 0.001; ****, *P* < 0.0001. act, activated; AF, Alexa Fluor; CTRL, control; GSK, glycogen synthase kinase-3; iso, isotype; ns, nonsignificant; PLA, proximity ligation assay.

Because the interaction between FH and T cells was not described previously, we started our investigation by evaluating whether FH can bind to T cells. We used primary human CD4^+^ and CD8^+^ T cells from peripheral blood, incubated in a serum-free medium supplemented with biotinylated FH. Biotinylated FH bound to CD4^+^ T cells in a dose-dependent manner, as detected by flow cytometry using Alexa Fluor 647-labeled streptavidin (Fig. 1A). To confirm the binding of FH to T cells and to investigate whether FH becomes internalized, CD4^+^ and CD8^+^ T cells were incubated with FH, and cell fractionation was carried out using a MEM-PER kit. An immunoblot showed that FH was found mainly in the membrane fraction of CD4^+^ (Fig. 1B) and CD8^+^ T cells (Supplementary Fig. S1A). The small amount of FH detected in the cytosol fraction was equal for cells incubated at 4°C and 37°C, which indicates passive uptake rather than active internalization.

To identify potential binding partners for FH on the surface of T cells, we profiled the binding activities of the C-terminal part of FH composed of CCP 19 to 20 with the human proteome array (HuProt). Using a highly stringent cut-off value, we identified the strongest binding partners. ICOS was the second strongest binder and the only receptor expressed on T cells among the identified binding partners of FH CCP19-20 (Fig. 1C). Because the HuProt microarray identifies interactions between purified proteins and does not provide information if a given protein can bind under more physiological conditions, the binding of FH to ICOS was confirmed by co-immunoprecipitation. Biotinylated FH–ICOS complexes, formed on the surface of T cells, were pulled down using streptavidin beads. The presence of ICOS in the complex was then detected with a specific antibody (Supplementary Fig. S1B).

To further confirm the interaction between FH and ICOS, we carried out an *in situ* PLA, which detects proteins in proximity (within 40 nm), most likely bound to each other. CD4^+^ T cells and activated CD4^+^ T cells were incubated in the presence or absence of FH. White dots indicating complexes of ICOS and FH could be detected in the samples containing CD4^+^ T cells and in higher numbers when the cells were activated (Fig. 1D and E; Supplementary Fig. S1C). The interaction between FH and ICOS was also confirmed by ELISA. CD4^+^ T cells incubated with biotinylated FH or biotinylated α1-AT, which was used as a negative control, were lysed and added to an anti-ICOS-coated plate. Bound FH was detected with streptavidin–HRP. FH bound to ICOS in a dose-dependent manner, whereas the negative control did not show any interaction (Fig. 1F). Additionally, we incubated CD4^+^ T cells with an antibody against ICOS, which resulted in a dose-dependent reduction of FH binding. Using the same concentrations of isotype control did not affect the interaction (Fig. 1G; Supplementary Fig. S1H).

It has been shown that ICOS is a major activator of PI3K signaling and that ICOS ligation with its known ligand (ICOSL) is associated with strong PI3K activity. Additionally, inhibition of PI3K and Akt signaling results in decreased ICOS function on Tregs (34). To define the functional effect of FH binding, Tregs remained unstimulated or stimulated with TransAct (CD3 and CD28 costimulation) or a combination of TransAct and FH. ICOS ligation by FH-induced stronger Akt phosphorylation than CD3 and CD28 alone (Fig. 1H and I). Blocking of ICOS with a specific mAb but not isotype control strongly reduced FH-induced Akt phosphorylation, indicating a specific contribution of ICOS-FH interaction in this event. To investigate this further, we analyzed another PI3K downstream molecule, glycogen synthase kinase-3. FH binding induced an increase in glycogen synthase kinase-3 phosphorylation, which was reduced by ICOS blocking (Fig. 1H and J). To compare FH with the known ICOSL, we determined the Kd of binding of these ligands to Tregs (Supplementary Fig. S1D and S1E). Next, we saturated the cells with FH, which resulted in decreased binding of ICOSL to Tregs (Supplementary Fig. S1F). Similarly, saturating ICOS with ICOSL affected FH binding in a comparable manner (Supplementary Fig. S1G), suggesting that FH and ICOSL might bind to the same site. Taken together, these data show that FH is a ligand for ICOS and the ligation leads to strong activation of PI3K/Akt-dependent signaling pathways in Tregs.

### FH increases the viability of CD4^+^ but not CD8^+^ T cells

**Figure 2.**
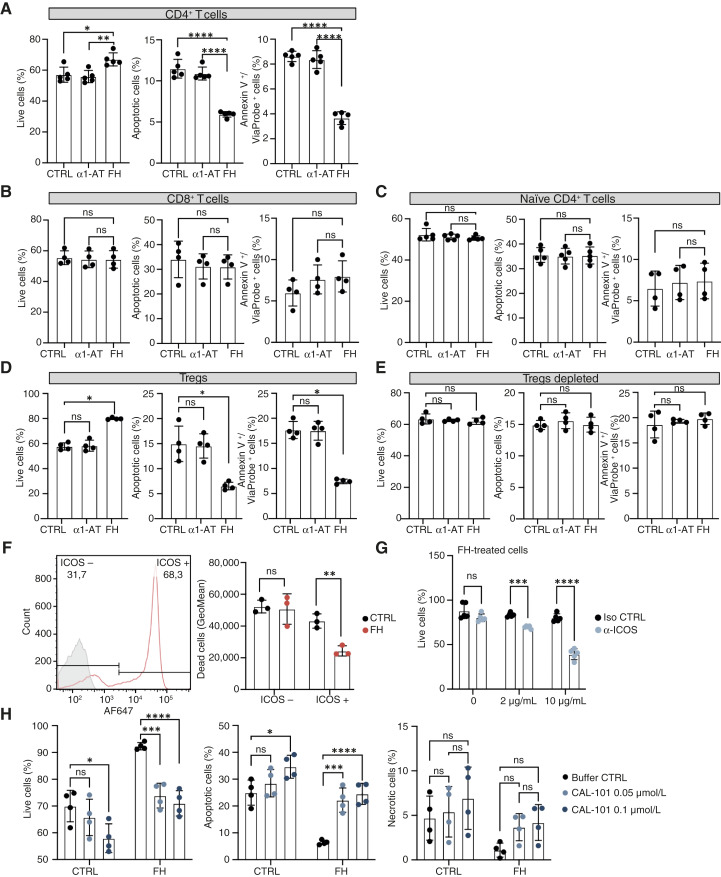
FH increases the viability of Tregs. FH-rendered increase in survival of CD4^+^ (**A**) but not CD8^+^ (**B**) or naïve CD4^+^ (**C**) T cells. FH increased survival of Tregs (**D**) and did not increase survival of Treg-depleted CD4^+^ T cells (**E**). Cells were incubated with medium, 150 μg/mL FH, or α1-AT for 7 days, assessed by Annexin V and Via-Probe staining. **F,** Gating of Tregs according to ICOS expression. FH rescues survival of ICOS^+^ but not ICOS^−^ Tregs incubated with medium alone and supplemented with 150 μg/mL FH. **G,** Incubation with 2 and 10 μg/mL of ICOS blocking antibody decreased the FH-rendered increase in Treg viability in a dose-dependent manner. **H,** The p110δ-specific PI3Kδ inhibitor CAL-101 negates the increase in Treg survival induced by 150 μg/mL of FH. Data are means ± SD of *n* = 4 (**A–D** and **H**), *n* = 3 (**F**), *n* = 5 (**G**) independent experiments. Statistical tests: two-way ANOVA with Tukey’s multiple comparison test (**A–C** and **F–H**), Kruskal–Wallis with Dunn’s multiple comparison test (**D** and **E**). *, *P* < 0.05; **, *P* < 0.01; ***, *P* < 0.001; ****P < 0.0001; AF, Alexa Fluor; CTRL, control; iso, isotype; ns, nonsignificant.

To investigate the functional consequences of the interaction between FH and ICOS, we specifically examined how FH affects cell viability. CD4^+^ and CD8^+^ T cells were treated with FH in a serum-free medium for 7 days. We assessed cell viability through flow cytometry, employing Annexin V and Via-Probe. We determined that FH treatment increased the viability of CD4^+^ T cells compared with the untreated cells; however, this increase was not evident in CD8^+^ T cells (Fig. 2A and B). Recognizing the heterogeneity of CD4^+^ T cells, we sought to discern if the effect of FH was universal among these cells or limited to specific subsets. To this end, we initially focused on the viability of naïve CD4^+^ T cells and we observed no statistically significant change in their survival (Fig. 2C). Subsequently, we shifted our focus to Tregs, which when incubated with FH for 7 days exhibited a significantly higher survival rate compared with both untreated cells and those treated with the negative control, α1-AT (Fig. 2D). Accordingly, the percentage of apoptotic and Annexin V^+^ Via-Probe^+^ cells were significantly reduced in cells treated with FH (Fig. 2D). To determine if Tregs were exclusively responsible for the observed increase in viability of CD4^+^ in response to FH, we isolated CD4^+^ cells and selectively depleted Tregs from this population. In the absence of Tregs, FH treatment did not confer any survival advantage to the remaining CD4^+^ T cells (Fig. 2E).

### FH effect on Treg viability is mediated by ICOS

Because the expression of ICOS on Tregs varies (35–37), we investigated if the increased viability of these cells upon FH binding was dependent on the expression of this receptor. To this end, we stimulated Tregs with FH for 7 days, after which we determined cell death with SYTOX Green and also measured ICOS expression. We found that the cell population with increased viability corresponded to the population expressing ICOS (Fig. 2F). When we excluded ICOS^+^ Tregs from the analysis, no statistically significant difference in viability was observed between FH-treated and untreated cells (Fig. 2F). Additionally, when the cells were pretreated with an ICOS blocking antibody, the changes in Treg viability induced by FH were diminished, whereas no changes were observed when the cells were pretreated with the isotype control (Fig. 2G).

### Increased viability of Tregs is mediated by the same pathway as ICOS signaling

We hypothesized that the mechanism by which FH increases T-cell viability is related to PI3Kδ signaling, which regulates lymphocyte metabolism, survival, proliferation, apoptosis, and migration. Activation of the PI3Kδ pathway by cell surface receptors, such as ICOS, which signals almost exclusively through PI3Kδ is directly mediated by class I isoforms, including p110δ (34). Therefore, we tested if the effect of FH on Tregs viability could be blocked by the p110δ-specific PI3Kδ inhibitor 5-fluoro-3-phenyl-2-[(S)-1-(9H-purin-6-ylamino)-propyl]-3H-quinazolin-4-one (CAL-101). Tregs incubated with FH exhibited higher viability than untreated cells but when Tregs were treated with CAL-101, the viability of FH-treated cells matched controls (Fig. 2H). The effect was detectable in the amount of live and apoptotic cells, whereas the number of Annexin V^+^ Via-Probe^+^ cells did not change significantly, indicating that the effect is not caused by the toxicity of the compound (Fig. 2H).

### FH enhances the immunosuppressive function of Tregs

**Figure 3.**
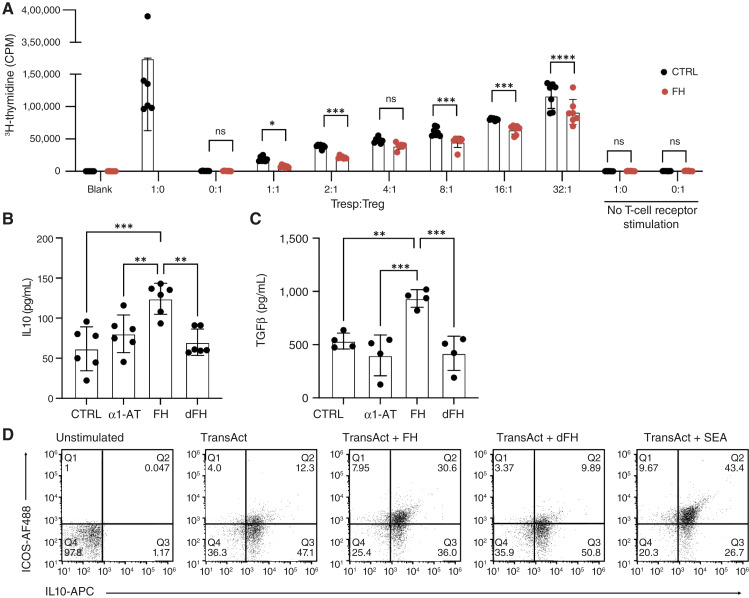
FH enhances the immunosuppressive function of Tregs. **A,** FH increases the immunosuppressive effect of Tregs. Tregs were incubated in medium only or supplemented with 150 μg/mL FH. CD4^+^ T cells were isolated from the same donor and cocultured with Tregs in the presence of CD2/CD3/CD28 MACSiBeads and ^3^H-thymidine. Secretion of IL10 (**B** and **D**) and TGFβ (**C**) by Tregs incubated in medium only, 150 μg/mL α1-AT, FH, or dFH. Cells were stimulated with anti-CD3 and anti-CD28 microbeads in the presence of IL2, and secreted cytokines were measured by ELISA after 48 hours (**B**) or 72 hours (**C**). **D,** Frequency of IL10-producing Tregs, either unstimulated, or stimulated for 16 hours with TransAct, TransAct + FH, TransAct + dFH, or TransAct + SEA, relative to ICOS expression, was assessed with IL10 catch. Data are means ± SD of *n* = 7 (**A**), *n* = 6 (**B**), *n* = 4 (**C**) independent experiments; representative plots of *n* = 5 (**D**) independent experiments. Statistical tests: two-way ANOVA (**A**) and one-way ANOVA (**B** and **C**) with Tukey’s multiple comparison test. *, *P* < 0.05; **, *P* < 0.01; ***, *P* < 0.001; ****, *P* < 0.0001. AF, Alexa Fluor; CPM, count per minute; CTRL, control; ns, nonsignificant; SEA, staphylococcal enterotoxin A; Tresp, responder CD4^+^ T cells.

The primary function of Tregs is to suppress excessive or abnormal immune responses to self and non-self antigens, thus maintaining immune homeostasis (38). We examined whether FH could enhance the cell-mediated immunosuppressive function of Tregs. Our analysis focused on the proliferative capacity of CD4^+^ T cells cultured with or without FH-treated Tregs in the presence of CD2/CD3/CD28 MACSiBeads and ^3^H-thymidine. We observed that although Tresp vigorously proliferated in the absence of Tregs, their proliferation was markedly suppressed in the presence of Tregs (Fig. 3A). FH preincubation enhanced this effect.

Tregs exert their immunosuppressive function via several mechanisms, such as the production of inhibitory cytokines, including IL10, IL35, and TGFβ (39). To investigate if these mediators are involved in the FH-induced increase in immunosuppression, Tregs were preincubated with FH, followed by stimulation with anti-CD3, and anti-CD28 microbeads in the presence of IL2. Analysis of supernatants by ELISA revealed that FH significantly increased the secretion of IL10 (Fig. 3B) and TGFβ (Fig. 3C). ICOS expression on expanded Tregs enables them to preferentially produce IL10 (40, 41). In line with this, patients with ICOS deficiency show a severe reduction in IL10 production, reinforcing the role of ICOS in IL10 secretion (42). To investigate if the FH-rendered increase in IL10 secretion was mediated via ICOS, Tregs were either nonstimulated or stimulated with TransAct in combination with FH, TransAct with dFH, or TransAct with positive control SEA. The capacity to produce IL10 was assessed by flow cytometry. The expression level of ICOS correlated with FH-induced IL10 secretion, whereas stimulation with dFH did not show a similar effect (Fig. 3D).

### FH knockdown is associated with a lower number of ICOS^+^ Tregs in a murine glioma model

**Figure 4.**
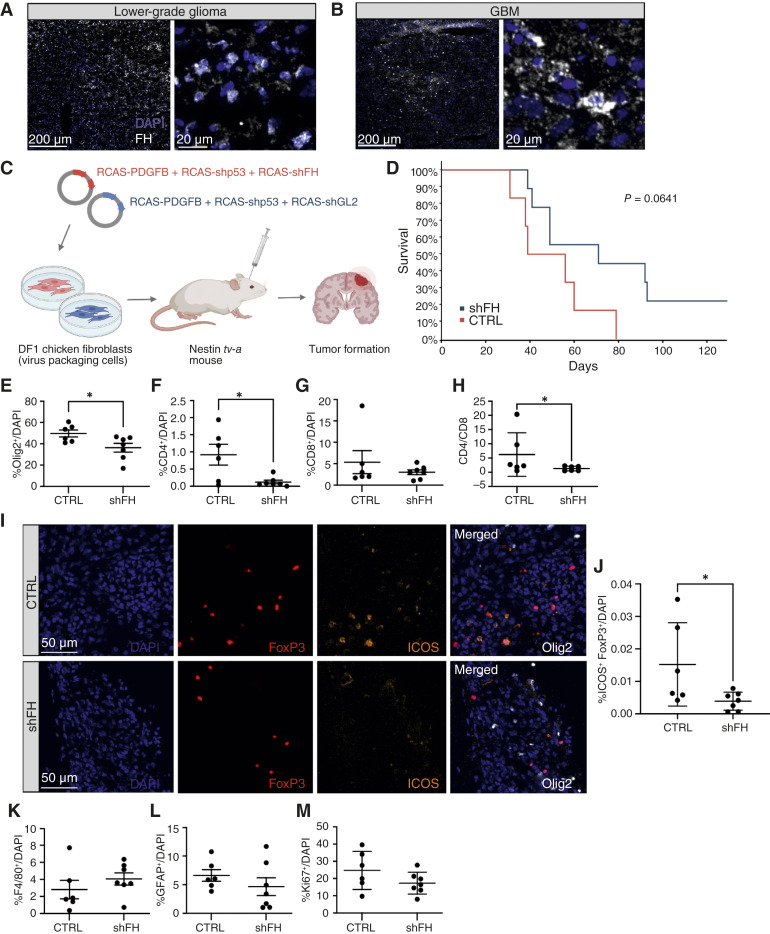
FH knockdown is associated with a lower number of ICOS^+^ Tregs in a murine glioma model. FH expression was investigated in murine gliomas generated using the RCAS/tv-a vectors to induce expression of *PDGFB* (**A** and **B**) and *shp53* (**B**). **C,** Schematic illustration of the mouse model. Increased survival (**D**), decrease in number of Olig2^+^ tumor cells (**E**), changes in infiltration of T cells (**F–J**), and macrophages (**K**) as well as number of astrocytes (**L**) and changes in proliferation of tumor cells (**M**) were investigated in GBM model with shRNA-induced FH knockdown. Vector targeting *Gl2* was used as a control. Statistical analysis: logrank test (**D**), Student *t* test with Welch correction (**E–G** and **J–M**), Student *t* test paired (**H**). Data are means ± SD of *n* = 15 (**D**), *n* = 13 (**E–H** and **J–M**) mice. *, *P* < 0.05; **, *P* < 0.01; ***, *P* < 0.001; ****, *P* < 0.0001. CTRL, control; Gl2, firefly luciferase; RCAS/tv-a, replication-competent ASLV long terminal repeat with a tv-a splice acceptor. (**C,** Created with BioRender.com.)

Tregs have been shown to play a detrimental role in glioma progression (43). To study the relevance of our findings in a tumor context, we tested the effect of FH knockdown on survival in a glioma mouse model. First, we investigated whether FH is expressed in murine gliomas. Glioma tumors were generated using the replication-competent ASLV long terminal repeat with a tv-a splice acceptor (RCAS/tv-a) system in Nestin/tv-a (Ntv-a) mice. *PDGFB* overexpression was used for induction of lower-grade glioma, and a combination of *PDGFB* and shRNA-mediated knockdown of *p53* were used to induce higher-grade glioma (GBM). We found that FH was expressed in both models (Fig. 4A and B), and no signal was detected in the negative controls (Supplementary Fig. S2E). To determine if FH can bind to Tregs in mice, we incubated primary mouse–derived Tregs with mouse FH (Supplementary Fig. S2A). Next, we isolated tumor cells from Ntv-a mice. After culturing for 7 days, we immunodepleted FH in the supernatant using magnetic beads coupled with anti-FH. When primary mouse Tregs were incubated in the supernatant derived from tumor cells, we observed that FH derived from tumor cells increased Treg survival, and this effect diminished as the concentration of FH was reduced (Supplementary Fig. S2B and S2C).

Next, we knocked down FH in tumor cells of GBM using shRNA and we employed shRNA targeting firefly luciferase (*Gl2*) as a negative control (Fig. 4C). We confirmed FH knockdown on the protein level by Western blot analysis of the supernatants derived from NIH3T3-tva cells (30), transfected with supernatants from the DF1-RCAS-shFH/DF-1-RCAS-shGl2 cells (Supplementary Fig. S2D). We observed that in this aggressive glioma model, FH knockdown showed a tendency to rescue survival (*P* = 0.06) and led to a significant reduction in glioma cell numbers [oligodendrocyte transcription factor 2 (Olig2)^+^; Fig. 4E]. Consistent with our previous findings, we could also observe higher infiltration of CD4^+^ (Fig. 4F), but not CD8^+^ T cells (Fig. 4G) in mice expressing FH in comparison to FH knockdown. Additionally, the ratio of CD4^+^ to CD8^+^ T cells, an indicator of an active immune response, was significantly decreased in FH knockdown mice (Fig. 4H). Furthermore, we could observe a significant decrease in ICOS^+^ Tregs indicating an important role of FH in sustaining these cells in the glioma microenvironment (Fig. 4I and J). Macrophage accumulation (F4/80^+^; Fig. 4K) and the number of astrocytes (GFAP^+^; Fig. 4L) were not significantly affected by FH knockdown.

To test whether FH may have a direct effect on tumor cells, we treated tumor cells from Ntv-a mice, PIGPC cells, with mouse FH. The proliferation (Supplementary Fig. S3A) and survival (Supplementary Fig. S3D–S3F) of the cells were not affected by FH, suggesting that the effect of FH on mouse survival was not because of a direct effect on tumor cells. These results were confirmed using mock or FH-transfected H4 cells (Supplementary Fig. S3B and S3G–S3I). Additionally, we could not detect a correlation between FH expression and KI67^+^ cells in our mouse model (Fig. 4M). To confirm these findings, we used data from the GEO database, which also showed a lack of significant correlation between Ki67 expression in tumor cells and FH expression in patients with glioma (Supplementary Fig. S3C).

### *ICOS* is associated with poor prognosis in human glioma

**Figure 5.**
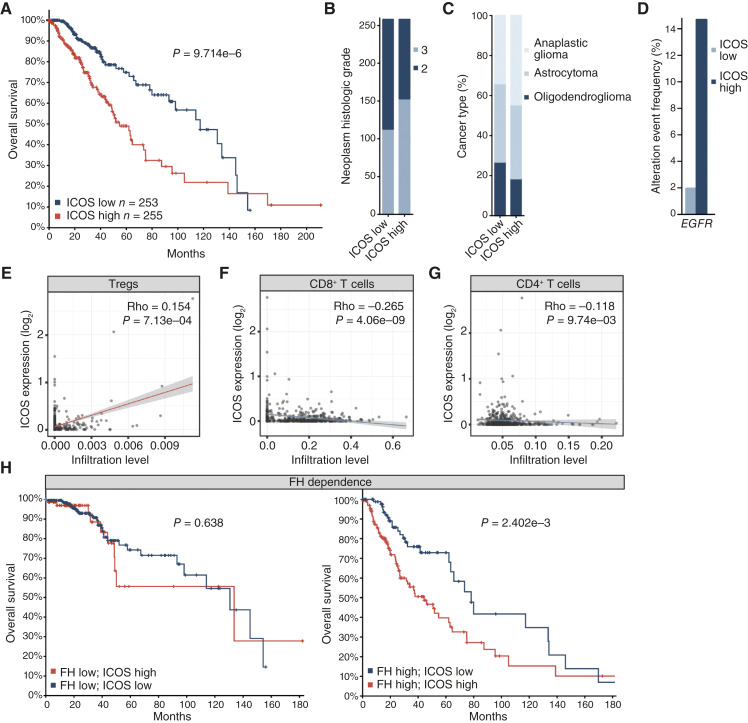
ICOS is associated with poor prognosis of patients with glioma. **A,** ICOS expression correlates with decreased survival of patients with glioma. Expression of ICOS correlates with neoplasm histological grade (**B**), cancer type (**C**), and *EGFR* mutations (**D**). ICOS positively correlates with infiltration of Tregs (**E**) and negatively with infiltration of CD8^+^ T cells (**F**) and CD4^+^ T cells (**G**) in lower-grade glioma. **H,** In a patient cohort stratified based on FH expression, ICOS correlates with worse survival only in the FH-high group. The survival estimates and correlation with other clinical parameters were analyzed in cBioPortal and based on the TCGA provisional dataset brain lower-grade glioma. The cases were set to include samples with mRNA data from *n* = 508 patients (**A–D** and **H**). Correlation analysis of the immune infiltration was performed using TIMER2.0, based on *n* = 516 samples from patients with lower-grade glioma. Statistical tests: logrank test (**A–H**), *χ*^2^ test (**B** and **C**), one-sided Fisher exact test (**D**), and Spearman’s rho correlation (**E–G**).

The expression of ICOS, an immune costimulatory molecule, has attracted a lot of attention in the context of solid and hematologic malignancies. However, despite insightful but sparse reports (44), the role of ICOS in glioma is still poorly understood. Using publicly available datasets and the cBioPortal search engine, we found that high expression of *ICOS* correlated with poor survival of patients with glioma (Fig. 5A). *ICOS* expression was also significantly linked with higher tumor grade (*P* = 1.59e−3; Fig. 5B), WHO histological subtype (*P* = 1.49e−3; Fig. 5C) and the presence of *EGFR* mutations (*P* = 4.67e−8; Fig. 5D). Additionally, we found a positive correlation between *ICOS* expression and the presence of Tregs (Fig. 5E), whereas a negative correlation was observed with CD8^+^ (Fig. 5F) and CD4^+^ T cells (Fig. 5G). Moreover, the correlation of ICOS with the survival of patients with glioma was observed solely in the subgroup with high levels of FH expression. Hence, the ICOS-related effect on patient survival was contingent upon the presence of FH (Fig. 5H).

### FH is expressed in human glioma and correlates with disease severity

**Figure 6.**
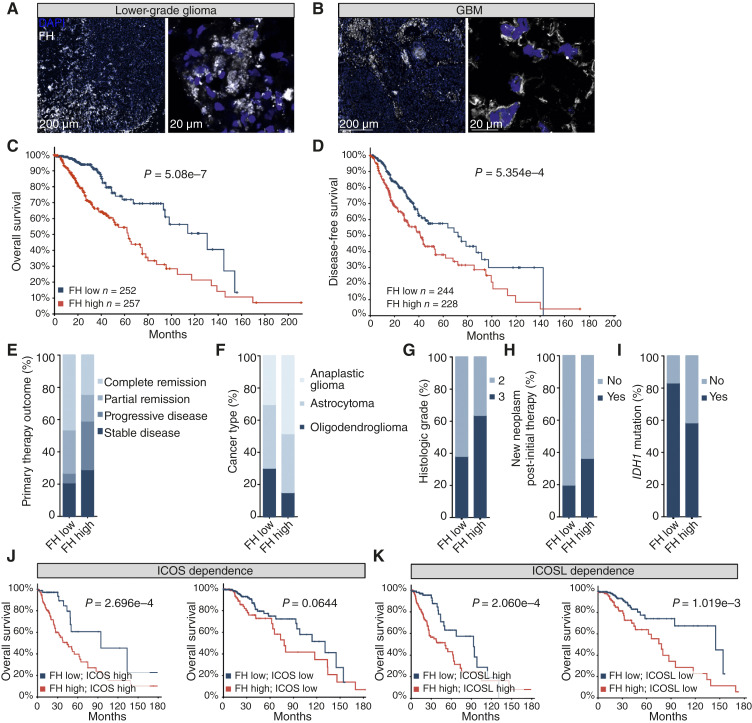
FH is expressed in human glioma and correlates with disease severity. FH is produced in human lower-grade glioma (**A**) and GBM (**B**). **C,** FH expression correlates with decreased overall survival (**C**) and disease-free survival (**D**) of patients with glioma. Expression of FH correlates with primary therapy outcome (**E**), cancer type (**F**), neoplasm histological grade (**G**), new neoplasm event post-initial therapy (**H**), and *IDH1* mutation rate (**I**). The worse survival rendered by FH is ICOS-dependent (**J**) but not dependent on ICOSL (**K**). The survival estimates and correlation with other clinical parameters based on mRNA and survival data of *n* = 509 (**C**, **J**, and **K**), *n* = 472 (**D**) patients from TCGA provisional dataset brain lower-grade glioma, analyzed with cBioPortal. Statistical tests: logrank test (**C**, **D**, **J**, and **K**), *χ*^2^ test (**E–I**). Representative picture of *n* = 2 (**A**), *n* = 3 patients (**B**).

Because FH was not previously known to be expressed in human gliomas, we analyzed samples from three GBM and two patients with astrocytoma, with ages ranging from 26 to 70 years (Supplementary Fig. S4A and S4B). The specificity of anti-FH was verified using paraffin-embedded pellets of mock or FH-transfected HEK 293 cells as described before (21). Our findings showed consistency with the data from the mouse model, revealing FH expression in lower-grade glioma and GBM (Fig. 6A and B).

To evaluate the correlation between *FH* expression in glioma and clinical outcomes, we used TCGA datasets. Interestingly, patients with higher transcriptional levels of FH had significantly shorter overall survival (Fig. 6C), and disease-free survival (Fig. 6D). We also found a significant association between the intensity of FH expression and several clinical parameters including primary therapy outcome (*P* = 1.06e−6; Fig. 6E), WHO histological subtype (*P* = 5.02e−6; Fig. 6F), recurrent disease (*P* = 8.34e−4; Fig. 6G), histological grade (*P* = 6.75e−8; Fig. 6H), and *IDH1* mutations (*P* = 3.96e−3; Fig. 6I).

### Worse prognosis associated with *FH* is dependent on *ICOS* but not *ICOSL* expression

To evaluate if the association of *FH* on patients’ survival was associated with ICOS interactions, we stratified the cohort by median *ICOS* expression. We discovered that high *FH* expression was significantly associated with worse prognosis in the *ICOS* high group, but not in the *ICOS* low group (Fig. 6J). To eliminate the possibility that the correlation between FH and ICOS was mediated by changes in the level of ICOSL, we stratified the cohort based on *ICOSL* expression. High *FH* expression was significantly associated with worse prognosis both in the *ICOSL* high and *ICOSL* low groups (Fig. 6K). However, the patients with either *FH* low and *ICOSL* high or patients with *FH* high and ICOSL low had comparable prognoses (Supplementary Fig. S5A and S5B).

### FH in glioma correlates with Treg occurrence and increased Treg viability

**Figure 7.**
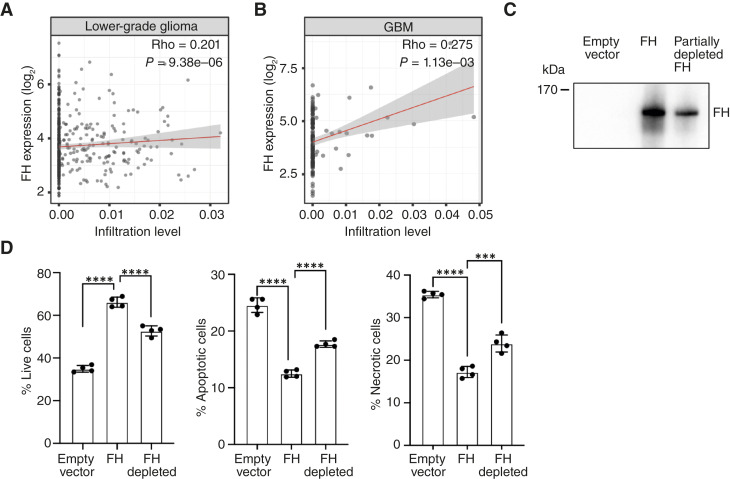
FH in glioma correlates with Treg occurrence and increased Treg viability. FH expression positively correlates with infiltration of Tregs in lower-grade glioma (**A**) and GBM (**B**). **C,** Western blot detecting FH in supernatants of FH-transfected glioma cell lines and depletion of FH with MRC-OX24-bound Dynabeads. **D,** Tregs incubated with supernatants of FH-transfected H4 glioma cell line exhibited increased survival, and the effect is weaker when FH is partially depleted. Correlation analysis of the immune infiltration was performed using TIMER2.0, based on *n* = 516 samples from lower-grade glioma and *n* = 599 samples from patients with GBM. Data are means ± SD of *n* = 4 (**D**) independent experiments. Representative blot of *n* = 4 (**C**) independent experiments. Statistical tests: Spearman’s rho (**A** and **B**), Kruskal–Wallis with Dunn’s multiple comparison test (**D**). ***, *P* < 0.001; ****, *P* < 0.0001.

Using Timer2.0 web resource, we showed that high FH expression was associated with the higher occurrence of Tregs in gliomas (Fig. 7A) and GBM (Fig. 7B). To investigate whether the increased presence of Tregs in the microenvironment could be associated with an FH-induced increase in the viability of these cells, we tested if FH derived from glioma cells would have the same effect on Tregs as plasma-purified FH. The H4 glioma cell line was transfected with cDNA coding for FH or the corresponding empty vector, and the expression of FH was determined using a Western blot analysis (Fig. 7C). The transfected cell line showed high levels of FH expression. Tregs treated for 7 days with supernatants from FH-transfected cells exhibited a higher percentage of live cells and a lower percentage of apoptotic and Annexin V^+^ Via-Probe^+^ cells (Fig. 7D). To determine if this effect was solely caused by FH or other factors in the supernatant altered concurrently with FH expression, we partially depleted FH in the supernatants using magnetic beads coupled with MRC-OX24 anti-FH (Fig. 7C). Reduced concentration of FH translated to decreased effect on Treg viability (Fig. 7D).

### FH correlates with key immunomodulatory molecules *IL10*, *TGFB*, and *IDO* in glioma

Because FH had an impact on cytokine secretion by Tregs, we investigated if this also occurred in glioma. Using cBioPortal, we discovered that *FH* expression was associated with higher mRNA transcription of *IL10* (Supplementary Fig. S6A) and *IL10* receptor alpha and beta (Supplementary Fig. S6B and S6C). The expression of *TGFB1* (Supplementary Fig. S6D) and *TGFB* receptors 1 and 2 (Supplementary Fig. S6E and S6F) also correlated with higher FH. The enzyme indoleamine 2,3-dioxygenase (IDO), crucial in the catabolism of tryptophan to kynurenine, has emerged as a pivotal factor in the tumor landscape. It has been reported that glioma cells can be induced to express IDO, a phenomenon potentially linked to tumorigenesis (45). Moreover, IDO has been shown to inhibit T-cell activation and proliferation (46) while regulating the recruitment (47), differentiation (48), and expansion and activation (49) of Tregs. In our dataset, *FH* expression positively correlated with *IDO1* (Supplementary Fig. S6G) and *IDO2* (Supplementary Fig. S6H) expression.

### *FH* expression correlates with immune checkpoints in glioma

Immune checkpoints are regulatory pathways that maintain self-tolerance and prevent autoimmunity, but they can be exploited by gliomas to evade the immune system’s surveillance. Targeting these immune checkpoints has emerged as a promising therapeutic strategy for gliomas, aiming to enhance antitumor immune responses and improve patient outcomes (50).

To further elucidate the role of FH in the glioma microenvironment, we used cBioPortal analysis of the dataset described above, and we revealed a positive correlation between *FH* and higher expression of the immune checkpoint *CTLA4* (Supplementary Fig. S6I). Although cytotoxic T-lymphocyte-associated protein 4 (CTLA-4) is not exclusive to Tregs, its expression is higher on Tregs than on other T-cell subsets. CTLA-4 is vital for the function of Tregs, and it inhibits the activation of effector T cells by outcompeting CD28 for its ligands, CD80 and CD86 (51). Additionally, we observed a correlation between *FH* expression and higher expression of the immune checkpoint molecules *TIM*3 (Supplementary Fig. S6J) and *LAG3* (Supplementary Fig. S6K), shown to render the glioma microenvironment immunosuppressive and associated with worse prognosis for patients with glioma (52–54). Although *FH* expression correlated with a lower level of *PD1*, the expression of its ligand, *PDL1* was upregulated (Supplementary Fig. S6L and S6M).

## Discussion

ICOS, expressed in primary human T cells, has emerged as a new target for cancer immunotherapy. In this study, we report that FH is a cancer cell-derived ligand for ICOS. The interaction of FH with ICOS is specific to Tregs and enhances their survival and activity. We show that FH is expressed in glioma and that high levels correlate with a worse prognosis and a higher occurrence of Tregs but not nonregulatory CD4^+^ T cells. This effect of FH on glioma development was confirmed in a mouse model.

It was recently shown, using glioma mouse models, that infiltration of CD8^+^ and CD4^+^ T cells is induced as soon as 10 days after intracranial tumor implantation. However, at day 15 the number of CD4^+^ T cells began to drop, demonstrating that the initial immune response was quickly replaced by T-cell anergy and immunosuppression (6). Additionally, although lymphopenia is often observed in the cervical lymph nodes of patients with glioma, spleen, and blood (3), levels of Tregs remain disproportionally high in the tumor microenvironment. The increased ratio of Tregs to other CD4^+^ T cells has been associated with the glioma grade and, subsequently, patient prognosis (55).

Tumor cells can manipulate the immune landscape by actively promoting the survival of immunosuppressive cells. It has been shown that Tregs cultured with tumor cell medium exhibit reduced expression of *BAX*, *BAK*, and *BIN* genes, responsible for apoptosis (56). In our experimental setup, human Tregs stimulated with plasma-purified and glioma cell-derived FH showed increased viability. We reported that FH had this effect only on ICOS^+^ T cells and that FH binds directly to this receptor. The suppressive function of Tregs was also enhanced by FH, which can be explained by the changes in TGFβ and IL10 release.

Although anti–PD-1 and anti–CTLA-4 targeting antibodies, so far, have been ineffective in the treatment of gliomas (57–60), combining known immune checkpoint blockades with novel targets remains a promising strategy. ICOS, essential to regulating T cells, represents a sensitive and strategic target. Currently, both agonistic and antagonistic monoclonal antibodies targeting ICOS are evaluated in clinical trials investigating different types of malignancies. In glioma, consistent with previous reports, our analysis showed a correlation between high ICOS expression, higher histological grade, and worse survival of patients with glioma. Additionally, we observed a positive correlation with *EGFR* mutations. Although the exact contribution of ICOS in glioma progression is not well understood, the immunosuppressive effect of ICOS may be attributed to increased activation and occurrence of Tregs (29, 46), and downregulation of these cells enhanced the impact of immunotherapy (50). In our cohort, we also observed a positive correlation between ICOS expression and Treg occurrence, whereas ICOS expression was negatively correlated with the occurrence of CD8^+^ and CD4^+^ T cells.

Conscious of difficulties in the translation of *in vitro* and *in vivo* results of immune therapy effectiveness to the clinical application, we utilized the RCAS/tv-a system to generate PDGFB-induced gliomas with or without p53 knockdown. PDGF signaling is frequently amplified in the proneural GBM molecular subtype and deemed a crucial initiating alteration in gliomagenesis and p53 tumor suppressor, is mutated in almost all astrocytomas, and is a subset of GBM. The combined effect of PDGFB overexpression and p53 depletion led to the consistent formation of high-grade tumors. It is worth noting that this model is not typically employed to study T cells, given the sparse infiltration of the lymphocyte population. Nonetheless, it provides an outstanding representation of the classic features of human GBM, including cellular pleomorphism, necrosis, microvascular proliferation, invasion along white matter tracts, and high proliferation rates (30, 61). Thus, we chose to prioritize the confidence in the biological relevance of the FH and ICOS effect demonstrated in this study, even in the microenvironment with low T-cell infiltration, above using a model that might offer a more substantial effect size.

Although the results from the *in vivo* experiments support and align with the conclusions of other experiments and analyses in this study, it is important to point out certain limitations inherent to this model. The FH knockdown alone does not provide direct proof that the effect of FH on glioma development is dependent on ICOS^+^ Tregs. Therefore, a more advanced model, such as combining FH knockdown with the ablation of ICOS^+^ Tregs in the glioma microenvironment, would be necessary to definitively rule out any direct effect of FH on glioma cells or other components of tumor microenvironment. Although we investigated some potential mechanisms such as the effect of FH on glioma cell proliferation or survival *in vitro*, we acknowledge the possibility that FH may have multiple modes of action waiting to be discovered using different models.

The upregulated expression of the only known ligand for ICOS, ICOSL, has been associated with poor prognosis of patients with glioma and alterations in cytokine profile in the tumor microenvironment (62). We observed the same association in our cohort. However, when we stratified the patients based on the FH expression, ICOS was associated with a worse prognosis only in the FH high group. That suggests that the role of FH is not redundant despite the likely presence of other ligands for ICOS.

Although the role of FH in the glioma microenvironment is still largely unknown, the production of FH by the glioma cell line CB193 was previously reported as a model to study complement biosynthesis [45]. Additionally, it was shown that FH is produced by the H2 GBM cell line and plays a role in protecting these cells against complement activation [46]. Our bioinformatic analysis of patients with glioma seems to confirm our *in vitro* and *in vivo* findings connecting FH expression with changes in the immunomodulatory molecules, including IL10, in the microenvironment. The elevated expression of IL10 in ICOS^+^ Tregs has been shown to correlate with the increased expression of several other markers, such as CTLA-4, Lag3, and PD-1, which are involved in the suppressive function of Tregs. These molecules, along with ICOS, contribute to the transcription of the IL10 gene, thus enhancing the inhibitory capabilities of Tregs (63). Our results were consistent with these findings, with the exception of PD-1, which was negatively correlated with high FH expression. On the other hand, the expression of PD-L1 was increased. It has been shown that human Tregs can promote immune suppression by inducing upregulation of PD-L1 on neighboring immune cells. This induction of PD-L1 by Tregs was reversed when the cells were blocked, highlighting a regulatory feedback loop between Tregs and the expression of PD-L1 in the immune system (64). Although this type of analysis does not allow us to determine if the FH-related changes were solely caused by the effect on Tregs, it provides correlative support for our hypothesized model. A recent study has also shown that nonenzymic IDO activity decreased the survival of glioma-bearing mice, increased the expression of FH, and increased the number of myeloid-derived suppressor cells and Tregs. Consistent with this study, we also observed a correlation between the expression of FH and IDO1 (65). Interestingly, the same study reports an effect of FH on macrophage maturation, arginase, and IL6 production, consistent with our previously published research about the role of macrophages in the breast cancer microenvironment (21). These data, in combination with our findings, provide an attractive connection between IDO1 and ICOS pathways and offer a deeper understanding of the function of these immune checkpoints. This is especially valuable because IDO1 is currently evaluated for the treatment of glioma.

In summary, we demonstrate that glioma-derived FH plays a role in the accumulation of Tregs in the glioma microenvironment by prolonging their survival. The effect is mediated by the binding of FH to ICOS and is limited to the ICOS^+^ Treg population. Moreover, FH expression is associated with worse survival of patients with glioma. The accumulation of Tregs in the glioma microenvironment may have considerable prognostic implications, and because FH binds directly to ICOS, a promising therapeutic target, we propose that FH expression should be scrutinized when considering the effectiveness of immunotherapies against glioma.

## Supplementary Material

Supplementary Figure 1 Supplementary figure 1. FH binds to T-cells via ICOS

Supplementary Figure 2 Supplementary figure 2. FH in Ntv-a mouse model (A) Mouse FH binds to mouse derived primary Tregs. Tregs were incubated for 2 h at 4oC with fluorescently labeled 25 or 100 μg/mL FH. The binding was detected using flow cytometry. (B) Western blot detecting FH in supernatants of tumor cells isolated from Ntv-a mice. FH was partially depleted with antibody against mouse FH bound to Dynabeads. (C) FH-rendered increase in survival of mouse Tregs. The cells were incubated with tumor cell derived supernatant and FH-depleted supernatant. After 7 days viability was assessed by Annexin V and Via-Probe staining. (D) FH was successfully knockdown with shFH. DF1 cells were transfected with three different shFH constructs and Gl2 shRNA. The knockdown of FH in NIH3T3 cells, transfected with supernatants from the DF1-RCAS-shFH/DF-1-RCAS-shGl2 cells was detected with goat anti-FH antiserum by western blot. (E) Control staining of mouse tumor sections. Samples were incubated with goat anti-FH antibody, goat IgG isotype control, and anti-goat IgG secondary antibody or only secondary antibody. Nucleus was stained with DAPI. Data are means ± *SD* of (A) n = 4, (C) n = 3 independent experiments. Representative blot (D) of n = 3 and picture (E) of n = 3 independent experiments. Statistical tests: two- Kruskal-Wallis with Dunn´s multiple comparison test (A, C). (*P<0.05, **P<0.01, ***P<0.001, ****P<0.0001, ns -, nonsignificant; CTRL, control.

Supplementary Figure 3 Supplementary figure 3. Direct effect of FH on glioma cells Proliferation of PIGPC cells treated with medium only, 25- or 100-μg/mL FH (A) and H4 mock or FH-transfected (B) was analyzed after 24, 48, 72 and 96 hours using CyQUANT assay. Data obtained at 24 hours was used for the normalization. (C) Correlation between FH and Ki67 gene expression in tumor cells from glioma patients, obtained from GEO database. Survival of PIGPC cells pretreated with 100 μg/mL FH (D-F) and H4 mock or FH-transfected (G-I) rendered apoptotic by treatment with 0.75 uM staurosporine for 24 h. Viability was assessed by Annexin V and Via-Probe staining. Data are means ± *SD* of (D, E, F) n = 4, (A, B, G, H, I) n = 3 independent experiments. Statistical tests: Two-way ANOVA with Bonferroni's multiple comparisons test (A, B), one-way ANOVA with Tukey´s multiple comparison test (D-I) Spearman’s rho correlation (C). (*P < 0.05, **P < 0.01, ***P < 0.001, ****P < 0.0001, ns, nonsignificant; CTRL, control.

Supplementary Figure 4 Supplementary figure 4. Staining control for human samples and additional information about patients (A) Histology and (B) clinical parameters of glioma patients. MGMT-methylation status; TMZ-temozolomide; Adj-adjusted. WT – wild type.

Supplementary Figure 5 Supplementary figure 5. FH, ICOSL and ICOS dependence on glioma patient survival. The survival data of *n* = 509 patients from TCGA provisional dataset brain lower-grade glioma, analyzed with cBioPortal. Statistical tests: Logrank Test.

Supplementary Figure 6 Supplementary figure 6. FH expression in glioma correlates with pro-tumorigenic markers
