# Supplementary material for: Complement Factor H Is an ICOS Ligand Modulating Tregs in the Glioma Microenvironment
Source: Cancer Immunol Res. 2024 Oct 8;13(1):122–38. doi: 10.1158/2326-6066.CIR-23-1092 (PMC11712038; doi:10.1158/2326-6066.CIR-23-1092)
Supplement: Supplementary Figure 1 — FH binds to T-cells via ICOS [file cir-23-1092_supplementary_figure_1_supps1.docx]

**
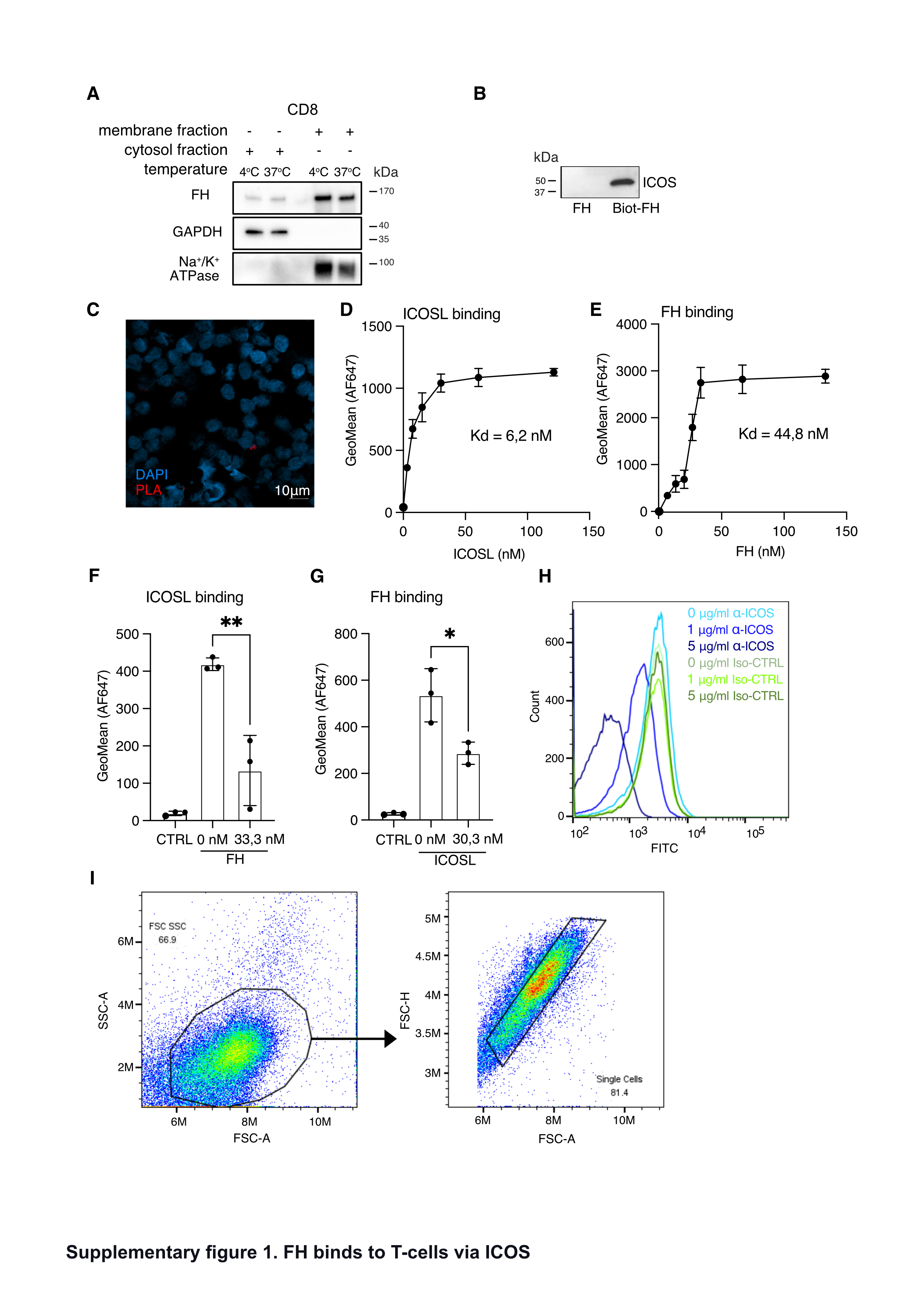
**

**Supplementary figure 1. FH binds to T-cells via ICOS**

(A) Fractionation detecting FH binding but not internalization into CD4^+^ and CD8^+^ T-cells, incubated with 150 μg/ml FH for 2 h in 4°C or 37°C. (B) Confirmation of FH binding to ICOS by co-immunoprecipitation of biotinylated-FH-ICOS complexes. CD4^+^ T-cells were incubated with FH or biotinylated FH for 2h in 37°C. Cells were lysed, and complexes were pulled down with streptavidin beads. Presence of ICOS was visualized using an anti-ICOS antibody. (C) Negative control for PLA. Activated CD4^+^ T-cells were incubated without FH for 2h at 37°C. PLA was performed with mouse anti-ICOS ab and goat anti-FH antiserum followed by anti-mouse and anti-goat probes. Determination of Kd value of ICOSL (D) and FH (E) binding to Tregs. 1x10^5^ Tregs were incubated for 2h at 4^o^C with various concentrations of fluorescently labeled ICOSL or FH. The binding was detected using flow cytometry. Assessment of binding of 3.3 nM of ICOSL (F) and 13.3 nM of FH (G) to 1x10^5^ Tregs preincubated for 2h at 4^o^C with 0 pM, 133 pM of FH or 0 pM, 12,1 pM of ICOSL. Binding of fluorescently labeled proteins was measured by flow cytometry. Unstained protein was used as a control. (H) Pre-incubation with anti-ICOS antibody reduced FH binding to CD4^+^ T-cells in a dose dependent manner. Isotype control was used at the same concentration, without affecting the binding. (I) Gating strategy for flow cytometry. Representative blot (A, B), histogram (H), dot plot (I) and picture (C) of n = 3 independent experiments. Data are means ± SD of (D-G) n = 3 independent experiments. Statistical tests: Kruskal-Wallis with Dunn´s multiple comparison test (F, G). Biot-FH – biotinylated FH; AF – Alexa Fluor; Kd – dissociation constant; CTRL – control.
