# Supplementary material for: Complement Factor H Is an ICOS Ligand Modulating Tregs in the Glioma Microenvironment
Source: Cancer Immunol Res. 2024 Oct 8;13(1):122–38. doi: 10.1158/2326-6066.CIR-23-1092 (PMC11712038; doi:10.1158/2326-6066.CIR-23-1092)
Supplement: Supplementary Figure 2 — FH in Ntv-a mouse model (A) Mouse FH binds to mouse derived primary Tregs. Tregs were incubated for 2 h at 4oC with fluorescently labeled 25 or 100 μg/mL FH. The binding was detected using flow cytometry. (B) Western blot detecting FH in supernatants of tumor cells isolated from Ntv-a mice. FH was partially depleted with antibody against mouse FH bound to Dynabeads. (C) FH-rendered increase in survival of mouse Tregs. The cells were incubated with tumor cell derived supernatant and FH-depleted supernatant. After 7 days viability was assessed by Annexin V and Via-Probe staining. (D) FH was successfully knockdown with shFH. DF1 cells were transfected with three different shFH constructs and Gl2 shRNA. The knockdown of FH in NIH3T3 cells, transfected with supernatants from the DF1-RCAS-shFH/DF-1-RCAS-shGl2 cells was detected with goat anti-FH antiserum by western blot. (E) Control staining of mouse tumor sections. Samples were incubated with goat anti-FH antibody, goat IgG isotype control, and anti-goat IgG secondary antibody or only secondary antibody. Nucleus was stained with DAPI. Data are means ± SD of (A) n = 4, (C) n = 3 independent experiments. Representative blot (D) of n = 3 and picture (E) of n = 3 independent experiments. Statistical tests: two- Kruskal-Wallis with Dunn´s multiple comparison test (A, C). (*P<0.05, **P<0.01, ***P<0.001, ****P<0.0001, ns -, nonsignificant; CTRL, control. [file cir-23-1092_supplementary_figure_2_supps2.docx]

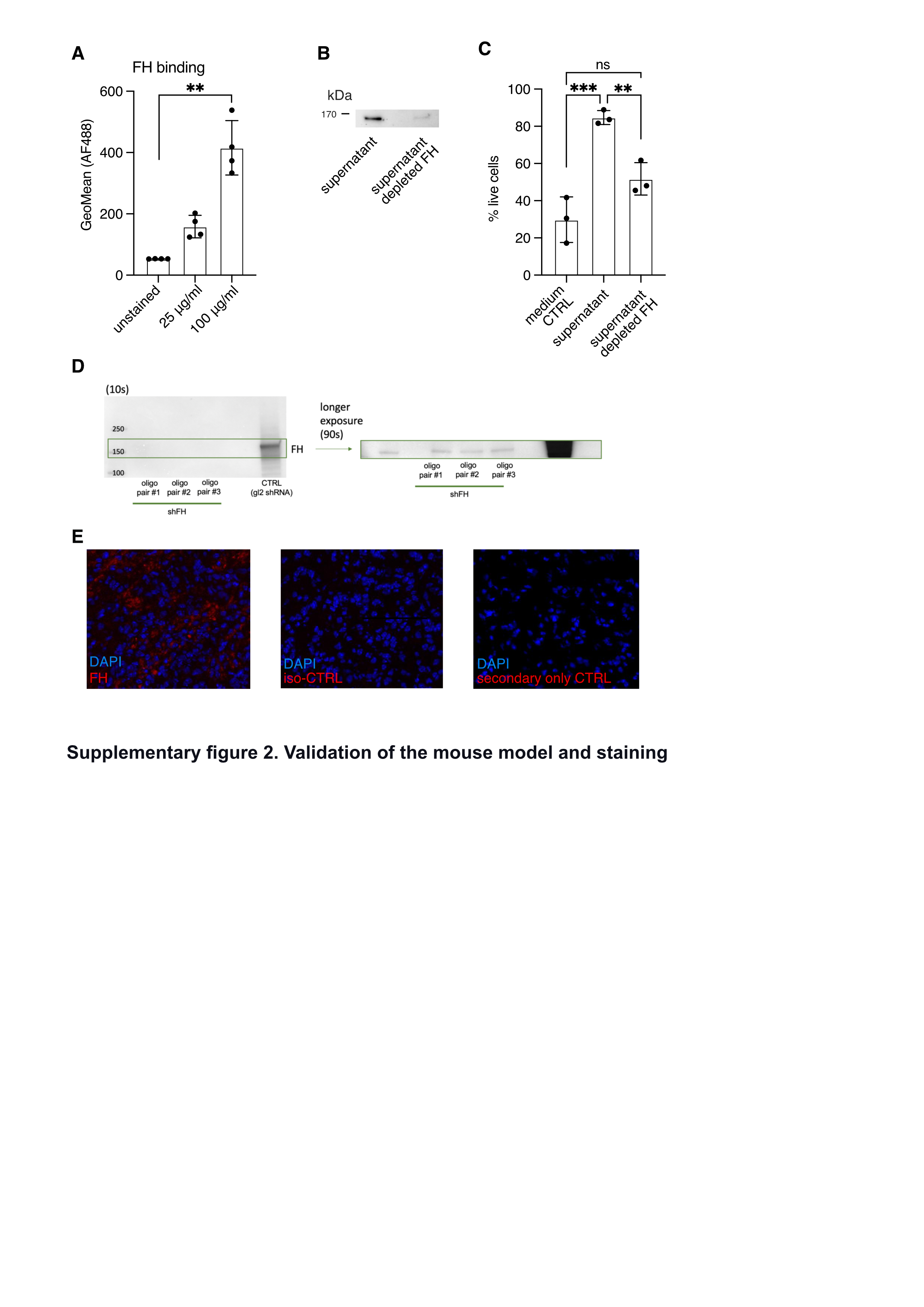


**Supplementary figure 2. FH in Ntv-a mouse model**

(A) Mouse FH binds to mouse derived primary Tregs. Tregs were incubated for 2h at 4^o^C with fluorescently labeled 25 or 100 μg/ml FH. The binding was detected using flow cytometry. (B) Western blot detecting FH in supernatants of tumor cells isolated from Ntv-a mice. FH-was partially depleted with antibody against mouse FH bound to Dynabeads. (C) FH-rendered increase in survival of mouse Tregs. The cells were incubated with tumor cell derived supernatant and FH-depleted supernatant. After 7 days viability was assessed by Annexin V and Via-Probe staining. (D) FH was successfully knockdown with shFH. DF1 cells were transfected with three different shFH constructs and Gl2 shRNA. The knockdown of FH in NIH3T3 cells, transfected with supernatants from the DF1-RCAS-shFH/DF-1-RCAS-shGl2 cells was detected with goat anti-FH antiserum by western blot**.** (E) Control staining of mouse tumor sections. Samples were incubated with goat anti-FH antibody, goat IgG isotype control, and anti-goat IgG secondary antibody or only secondary antibody. Nucleus was stained with DAPI. Data are means ± SD of (A) n = 4, (C) n = 3 independent experiments. Representative blot (D) of n=3 and picture (E) of n=3 independent experiments. Statistical tests: two- Kruskal-Wallis with Dunn´s multiple comparison test (A, C). (**P<*0.05, ***P<*0.01, ****P<*0.001, ****P<0.0001, ns - non-significant; CTRL – control.
