# Supplementary material for: Complement Factor H Is an ICOS Ligand Modulating Tregs in the Glioma Microenvironment
Source: Cancer Immunol Res. 2024 Oct 8;13(1):122–38. doi: 10.1158/2326-6066.CIR-23-1092 (PMC11712038; doi:10.1158/2326-6066.CIR-23-1092)
Supplement: Supplementary Figure 3 — Direct effect of FH on glioma cells Proliferation of PIGPC cells treated with medium only, 25- or 100-μg/mL FH (A) and H4 mock or FH-transfected (B) was analyzed after 24, 48, 72 and 96 hours using CyQUANT assay. Data obtained at 24 hours was used for the normalization. (C) Correlation between FH and Ki67 gene expression in tumor cells from glioma patients, obtained from GEO database. Survival of PIGPC cells pretreated with 100 μg/mL FH (D-F) and H4 mock or FH-transfected (G-I) rendered apoptotic by treatment with 0.75 uM staurosporine for 24 h. Viability was assessed by Annexin V and Via-Probe staining. Data are means ± SD of (D, E, F) n = 4, (A, B, G, H, I) n = 3 independent experiments. Statistical tests: Two-way ANOVA with Bonferroni's multiple comparisons test (A, B), one-way ANOVA with Tukey´s multiple comparison test (D-I) Spearman’s rho correlation (C). (*P < 0.05, **P < 0.01, ***P < 0.001, ****P < 0.0001, ns, nonsignificant; CTRL, control. [file cir-23-1092_supplementary_figure_3_supps3.docx]

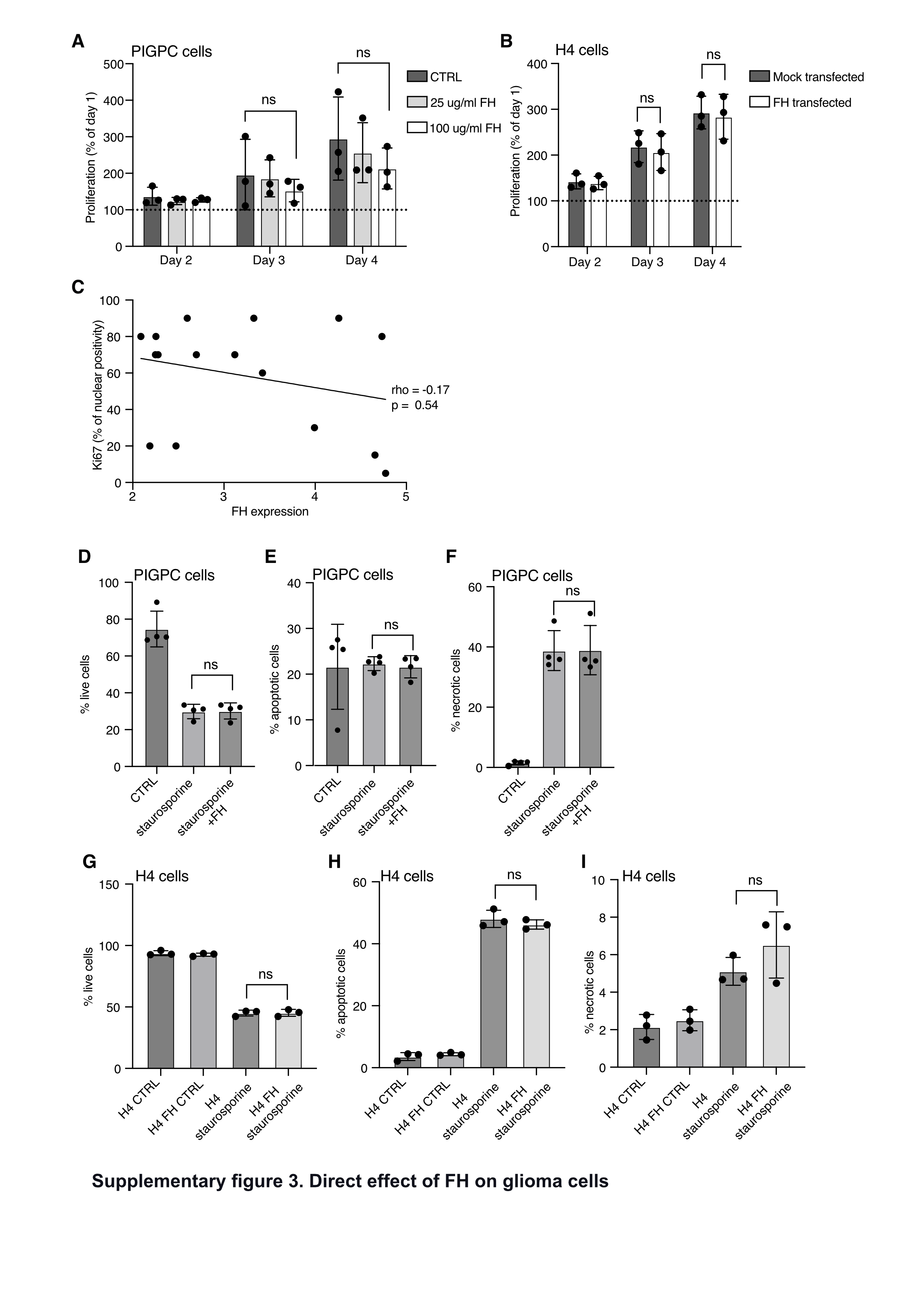


**Supplementary figure 3. Direct effect of FH on glioma cells**

Proliferation of PIGPC cells treated with medium only, 25 or 100 μg/ml FH (A) and H4 mock or FH-transfected (B) was analyzed after 24, 48, 72 and 96 hours using CyQUANT assay. Data obtained at 24 hours was used for the normalization. (C) Correlation between FH and Ki67 gene expression in tumor cells from glioma patients, obtained from GEO database. Survival of PIGPC cells pretreated with 100 μg/ml FH (D-F) and H4 mock or FH-transfected (G-I) rendered apoptotic by treatment with 0.75 uM staurosporine for 24h. Viability was assessed by Annexin V and Via-Probe staining. Data are means ± SD of (D, E, F) n = 4, (A, B, G, H, I) n = 3 independent experiments. Statistical tests: Two-way ANOVA with Bonferroni's multiple comparisons test (A, B), one-way ANOVA with Tukey´s multiple comparison test (D-I) Spearman’s rho correlation (C). (**P<*0.05, ***P<*0.01, ****P<*0.001, ****P<0.0001, ns - non-significant; CTRL – control.
