# Supplementary material for: Complement Factor H Is an ICOS Ligand Modulating Tregs in the Glioma Microenvironment
Source: Cancer Immunol Res. 2024 Oct 8;13(1):122–38. doi: 10.1158/2326-6066.CIR-23-1092 (PMC11712038; doi:10.1158/2326-6066.CIR-23-1092)
Supplement: Supplementary Figure 4 — Staining control for human samples and additional information about patients (A) Histology and (B) clinical parameters of glioma patients. MGMT-methylation status; TMZ-temozolomide; Adj-adjusted. WT – wild type. [file cir-23-1092_supplementary_figure_4_supps4.docx]

**Supplementary figure 4. Staining control for human samples and additional information about glioma patients**

(A) Histology and (B) clinical parameters of glioma patients.

MGMT-methylation status; TMZ-temozolomide; Adj-adjusted. WT – wild type.
