# Supplementary material for: Complement Factor H Is an ICOS Ligand Modulating Tregs in the Glioma Microenvironment
Source: Cancer Immunol Res. 2024 Oct 8;13(1):122–38. doi: 10.1158/2326-6066.CIR-23-1092 (PMC11712038; doi:10.1158/2326-6066.CIR-23-1092)
Supplement: Supplementary Figure 5 — FH, ICOSL and ICOS dependence on glioma patient survival. The survival data of n = 509 patients from TCGA provisional dataset brain lower-grade glioma, analyzed with cBioPortal. Statistical tests: Logrank Test. [file cir-23-1092_supplementary_figure_5_supps5.docx]

**
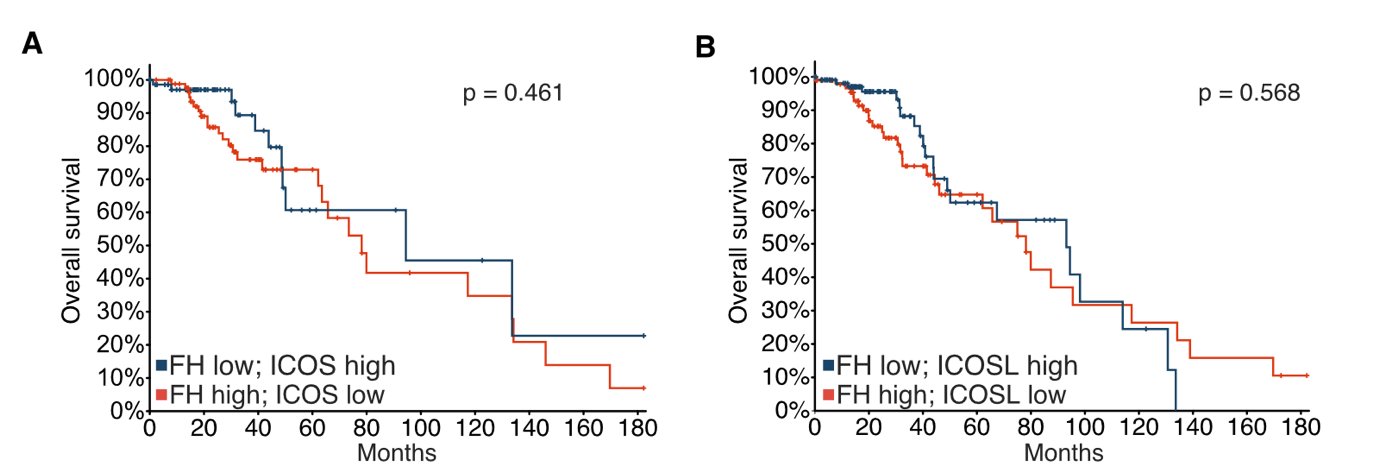
**

**Supplementary figure 5. FH, ICOSL and ICOS dependence on glioma patient survival.**

The survival data of n = 509 patients from TCGA provisional dataset brain lower-grade glioma, analyzed with cBioPortal. Statistical tests: Logrank Test.
