# Supplementary material for: Complement Factor H Is an ICOS Ligand Modulating Tregs in the Glioma Microenvironment
Source: Cancer Immunol Res. 2024 Oct 8;13(1):122–38. doi: 10.1158/2326-6066.CIR-23-1092 (PMC11712038; doi:10.1158/2326-6066.CIR-23-1092)
Supplement: Supplementary Figure 6 — FH expression in glioma correlates with pro-tumorigenic markers [file cir-23-1092_supplementary_figure_6_supps6.docx]

**
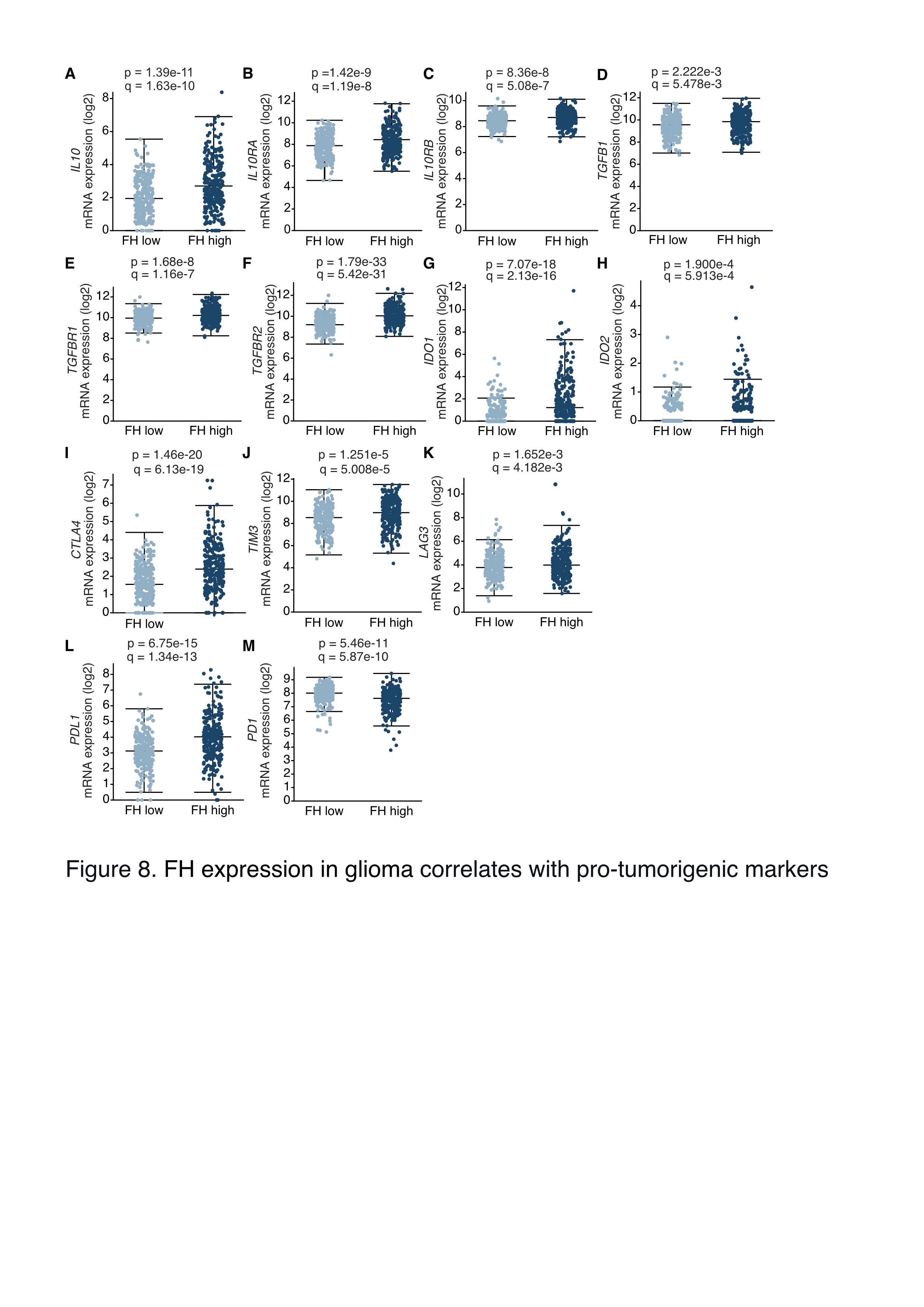
**

**Supplementary figure 6. FH expression in glioma correlates with pro-tumorigenic markers**

RNA-sequencing data (n = 509 patients) from TCGA provisional dataset brain lower-grade glioma, analyzed with cBioPortal, FH expression correlates with expression of *IL10* (A), IL10 receptor (B, C), *TGFB1* (D), and TGFB1 receptors (E, F), *IDO1* (G), and *IDO2* (H), *CTLA4* (I), *TIM3* (J), *LAG3* (K), *PDL1* (L), *PD1* (M). Student´s t-test (p-value), Benjamini-Hochberg procedure (q-value). TCGA - the cancer genome atlas.
